# Assessment of the Potential Diagnostic Value of Serum p53 Antibody for Cancer: A Meta-Analysis

**DOI:** 10.1371/journal.pone.0099255

**Published:** 2014-06-09

**Authors:** Jun Zhang, Zhiwei Xu, Lingxiang Yu, Meilan Chen, Ke Li

**Affiliations:** 1 Department of Preventive Medicine, Shantou University Medical College, Shantou, Guangdong, China; 2 Department of Medical Quality Control, 302 PLA hospital, Beijing, China; 3 Department of Hepatobiliary Surgery, 302 PLA hospital, Beijing, China; Sanjay Gandhi Medical Institute, India

## Abstract

**Background:**

Mutant p53 protein over-expression has been reported to induce serum antibodies against p53. We assessed the diagnostic precision of serum p53 (s-p53) antibodies for diagnosis of cancer patients and compared the positive rates of the s-p53 antibody in different types of cancers.

**Methods:**

We systematically searched PubMed and Embase, through May 31, 2012. Studies were assessed for quality using QUADAS (quality assessment of studies of diagnostic accuracy). The positive likelihood ratio (PLR) and negative likelihood ratio (NLR) were pooled separately and compared with overall accuracy measures using diagnostic odds ratios (DORs) and Area under the curve(AUC). Meta regression and subgroup analyses were done, and heterogeneity and publication bias were assessed.

**Results:**

Of 1089 studies initially identified, 100 eligible studies with 23 different types of tumor met the inclusion criteria for the meta-analysis (cases = 15953, controls = 8694). However, we could conduct independent meta analysis on only 13 of 36 types of tumors. Approximately 56% (56/100) of the included studies were of high quality (QUADAS score≥8). The summary estimates for quantitative analysis of serum p53 antibody in the diagnosis of cancers were: PLR 5.75 (95% CI: 4.60–7.19), NLR 0.81 (95%CI: 0.79–0.83) and DOR 7.56 (95% CI: 6.02–9.50). However, for the 13 types of cancers on which meta-analysis was conducted, the ranges for PLR (2.33–11.05), NLR (0.74–0.97), DOR (2.86–13.80), AUC(0.29–0.81), and positive rate (4.47%–28.36%) indicated significant heterogeneity. We found that breast, colorectal, esophageal, gastric, hepatic, lymphoma, lung and ovarian cancer had relatively reasonable diagnostic accuracy. The remaining results of the five types of cancers suggested that s-p53 antibody had limited value.

**Conclusions:**

The current evidence suggests that s-p53 antibody has potential diagnostic value for cancer, especially for breast, colorectal, esophageal, gastric, hepatic, lymphoma, lung and ovarian cancer. The results showed that s-p53 antibody had high correlation with cancers.

## Introduction

Cancer is the second leading cause of death following heart disease, accounting for 23% of all deaths [1]. From 2007 to 2008, the age-standardized cancer death rate decreased 1.5%, from 178.4 (per 100,000) to 175.8 [1] Despite decreases in the cancer death rates in high-resource countries, such as the United States, the number of cancer cases and deaths is projected to more than double worldwide over the next 20–40 years [2]. By 2030, it is projected that there will be 26 million new cancer cases and 17 million cancer deaths per year. The projected increase will be driven largely by the growth and aging of populations, and low-medium-resource countries will be the most affected [2]. Furthermore, the early stages of cancer are usually asymptomatic, and the prognosis of this disease is unfavorable in spite of advances in therapies. Cancer has long been recognized as a multi-step process that involves not only genetic changes conferring growth advantage, but also factors that disrupt regulation of growth and differentiation [3]. It is possible that some of these factors could be identified with the aid of auto-antibodies arising during tumorigenesis. Mutations in the tumor suppressor gene p53 are the most commonly observed genetic abnormalities in human cancers [4]. The protein product of the p53 gene is a nuclear phosphoprotein expressed in normal cells. In the serum of healthy subjects, the presence of p53 protein and anti-p53 antibodies are extremely rare [5]. Mutations in this gene cause an accumulation of non-functional proteins, due to increased stability and a longer half-life of several hours compared with the 20 min half-life for wild-type p53 [5]. The accumulated protein then acts as an antigen, with subsequent development of antibodies (anti-p53 antibodies), which are detectable in tissues, sloughed cells, blood, and other body fluids [5]. With the development of molecular biotechnology, a highly specific autoantibody response in systemic autoimmune diseases generally predicts the biologic phenotype of the disease, making autoantibodies clinically valuable and diagnostically useful [6]. Although current diagnostic procedures (pathologic examinations of resected specimens) improve the accuracy of the diagnosis, such procedures are often invasive, unpleasant, inconvenient and expensive. Hence, there is a great need for identification of novel non-invasive diagnostic methods for tumor detection. A large number of studies on the potential diagnostic value of serum p53 antibody for a variety of cancers have been published and have reported varying results. Our objective was to obtain the best estimates of the diagnostic accuracy of serum p53 (s-p53) antibody for detection of cancers, and to make comparisons about the diagnostic value of s-p53 antibody in different types of cancers by performing a systematic review and meta-analysis.

## Materials and Methods

### Search strategy and study selection

We did a systematic review of original articles that analyzed the diagnostic role of s-p53 antibody in patients with cancer, without language restriction. We identified 1090 articles from a search of PubMed and EMBASE databases using the search terms ‘neoplasm’, ‘blood OR serum’, ‘seropositive OR serum antibody’, ‘p53 or TP53’. No start data limit was applied. Details of the search strategy are shown in Table S2 in file S1. Articles were also identified by use of the related articles function in PubMed, and the references of identified articles were searched manually.

Two reviewers (J Zhang and ZW Xu) independently inspected the title and abstract of each citation to identify those studies that were likely to report the diagnostic value of serum p53 (s-p53) antibody, and then obtained the full text. Disagreements about study selection were resolved by consensus. The full text was retrieved for articles that could not be excluded based on title and abstract to determine inclusion. Inclusion criteria for the primary studies were as follows: (i) participants: all cases must have been diagnosed by pathologic examination of biopsied specimens, serum must have been collected for anti-p53 analysis before any treatment, e.g. chemotherapy or radiotherapy, and controls were without other cancers, (ii) index test: studies evaluated the diagnostic value of s-p53 antibody in cancer patients, (iii) outcome: studies reported the positive values of the cases and controls, and the results of an individual study on diagnostic accuracy could be summarized in a 2×2 table, (iv) study design: No restrictions were made with respect to study design (cross sectional, case control, cohort study) or data collection (prospective or retrospective). To avoid duplicate data, we identified articles that included the same group of patients by reviewing inter-study similarity in the country in which the study was done, investigators in the study, source of patients, recruitment period, and inclusion criteria. When the same investigators reported results obtained on the same group of patients in several publications, only the largest series was included in the analysis.

### Assessment of methodological quality

Two dependent reviewers (J Zhang and ZW Xu) used 11 items of published QUADAS (quality assessment for studies of diagnostic accuracy) guidelines as a tool to assess the included studies, and disagreements were resolved by consensus. The 11 items were recommended by the Cochrane Collaboration Methods Group on screening and diagnostic tests [7]. The items received a score of “1” if the item score was “yes” and aggregate scores were 11. Items included covered patient spectrum, reference standard, disease progression bias, verification bias, review bias, clinical review bias, incorporation bias, test execution, study withdrawals, and indeterminate results. The QUADAS tool is presented together with guidelines for scoring each of the items included in the tool.

### Data extraction and management

The primary reviewer (J Zhang) performed the preliminary extraction of data from each selected study using a standard form. Similarly, a second reviewer (Zhiwei Xu) also extracted the data to be used in the meta-analysis using the same form. Inter-reviewer discrepancies were resolved by discussion. The following characteristics studies were extracted: (i) basic information: conductor, study ID and study details (first author, year of publication, country of study, tumor type), (ii) study eligibility: based on inclusion/exclusion criteria to assess again and to record the reason for the excluded studies, (iii) methods of the study characteristics: participants' inclusion/exclusion criteria, ethnicity, disease stage, histology stage, standard reference, type of control, (iv) index tests: the extraction time and storage temperature of the sample, assay method, cut-off value, blind (single-blind or doubled-blind), a detailed report of the assay procedure, (v) outcome: the positive value of the cases and controls, and other comparison data (e.g. mean age, sex ratio, smoking, drinking) between cases and controls. We recorded the data according to the different types of cancers. If data from any of the above categories were not reported in the primary article, items were treated as “not reported”. We did not contact the authors for further details.

### Statistical analyses

We used standard methods recommended for meta-analysis of diagnostic test evaluations [8]. Statistical analysis was based on the following steps. 1) Presentation of the results of individual studies. Each study was presented with background information (year of publication, country, selection of patients and methodological characteristics). 2) Searching for the presence of heterogeneity. When different studies had largely different results, this may result from either random error or heterogeneity due to differences in clinical or methodological characteristics of the studies. A chi-square test was used to statistically test the presence of heterogeneity in study results. 3) Testing of the presence of cut-off threshold effects. Estimates of diagnostic accuracy differ if not all studies used the same cut-off point for a positive test result or for the reference standard. Variation in the parameters of accuracy may be partly due to variation in cut-off point. We tested for the presence of a cut-off point effect between studies by calculating the Spearman correlation coefficient between sensitivity and specificity of all included studies. 4) Dealing with heterogeneity. 5) Statistical pooling. The positive likelihood ratio (PLR), negative likelihood ratio (NLR) and their 95% confidence intervals (CI) were calculated using a random effects model based on the work of Der Simonian and Laird [9]. The likelihood ratio incorporates both the sensitivity and specificity of the test, and provides a direct estimate of how much a test result will change the odds of having a disease [10]. The PLR indicates how much the odds of the disease increase when a test is positive, and the NLR indicates how much the odds of the disease decrease when a test is negative [10]. Likelihood ratios of >10 or <0.1 generate large and often conclusive shifts from pretest to posttest probability (indicating high accuracy) [10]. According to Honest and Khan [11], sensitivity and specificity are considered inappropriate for meta-analyses, as they do not behave independently when they are pooled from various primary studies to generate separate averages. The accuracy measure used was the diagnostic odds ratio (DOR) computed by the Moses' constant of linear model, which indicates the change in diagnostic performance of the test under study per unit increase in the covariant [12]. The DOR is a single indicator of test accuracy that combines the sensitivity and specificity data into a single number [13]. DOR values range from 0 to infinity, with higher values indicating better discriminatory test performance (higher accuracy) [13]. A DOR of 1.0 indicates that a test does not discriminate between patients with the disorder and those without it [13]. Summary receiver operating characteristic curves were used to summarize overall test performance, and the area under the SROC curve (AUC) was calculated. The SROC curve has been recommended to represent the performance of a diagnostic test, based on data from meta-analysis, and the area under the SROC curve (AUC) is not only useful to summarize the curve, but also quite robust to heterogeneity [14], [15]. A prior study showed that to demonstrate excellent accuracy, the AUC should be in the region of 0.97 or above [16]. An AUC of 0.93 to 0.96 is very good; 0.75 to 0.92 is good. An AUC less than 0.75 can still be reasonable, but the test has obvious deficiencies in its diagnostic accuracy. The potential problem associated with sensitivities and specificities of 100% are solved by adding 0.5 to all cells of the diagnostic 2×2 table [8]. It means that we add 0.5 to each cell in only studies with zero cells.

We used a chi-squared test to detect statistically significant heterogeneity. Between-study heterogeneity was assessed using I^2^, according to the formula: I^2^ = 100%×(Cochran Q-degrees of freedom)/Cochran Q [17]. To assess cut-off threshold effects, the relationship between sensitivity and specificity was evaluated by using the Spearman correlation coefficient r. Possible sources of heterogeneity were investigated by meta-regression, which used a generalization of the Littenberg and Moses linear model weighted by the inverse of the variance [11]. Also, we conducted subgroup analysis. In order to evaluate the statistical outcome validity, we detected the pooled outcome by sensitivity analysis. Since publication bias is of concern for meta-analysis of diagnostic studies, we tested for the potential presence of this bias using funnel plots [18]. Publication bias is assessed visually by using a scatter plot of the inverse of the square root of the effective sample size (1/ESS1/2) versus the diagnostic log odds ratio (lnDOR) which should have a symmetrical funnel shape when publication bias is absent [19]. Formal testing for publication bias may be conducted by a regression of the lnDOR against 1/ESS1/2, weighting by ESS [19], with p<0.05 for the slope coefficient indicating significant asymmetry. All analyses were undertaken using Meta DiSc statistical software (version 1.4; Ramon y Cajal Hospital, Madrid, Spain) [20] and Stata SE12.0 software (Stata Corporation).

## Results

### Search results and study characteristics

**Figure 1:**
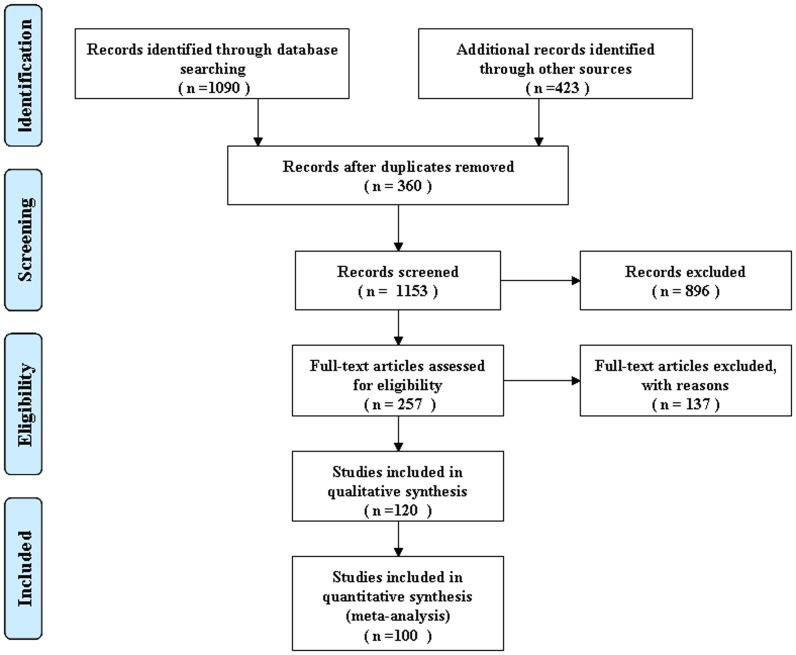
Flow chart of study selection by using electronic database.

Abstracts and titles of 1090 primary studies with 23 different types of cancer were identified for initial review using the search strategies. After reading the titles and abstracts, 896 unrelated articles were excluded, resulting in the acquisition of 257 full-texts on the role of s-p53 antibody in the diagnosis of cancer (Figure 1). Of these publications, 66 articles, including a review and case report, were excluded because they provided insufficient information. An additional 38 were excluded because there was no control, and 33 studies were excluded because they focused on the p53 gene and p53 protein, but did not detect s-p53 antibody. As a consequence, only 120 publications were considered to be eligible for inclusion in the analysis. However, 20 studies with controls were subsequently excluded because they did not allow the calculation of sensitivity or specificity. Finally, the remaining 100 articles (see Reference 1-100 in File S1), based on cases with cancer and controls without cancer, were available for meta-analysis, and the diagnostic characteristics of these studies, along with QUADAS scores, are outlined in Table S1 (a,b) in file S1. These studies followed several different characteristics. The studies included were conducted in different countries, 58 of 100 studies were conducted in western countries, 39 in Asia, one (see Reference 69 in File S1) in Brazil, one (see Reference 85 in File S1) in Nigeria, and one (see Reference 91 in File S1) in a multicenter trial. The publication years of the eligible studies ranged from 1987 to 2011. 15 studies chose consecutive patients, three(see Reference 1,77,96 in File S1) chose random patients, and 82 did not report related information. Only three studies(see Reference 63,73,100 in File S1) were prospective studies. 41 studies provided TNM stage and 19 provided histology stage. 54 of the studies included healthy volunteers as a control, 20 studies included healthy volunteers and patients with benign disease as controls, 20 studies included only the benign disease control, and the remaining 6 (see Reference 3,7,10,37,61,97 in File S1)did not report the type of the control.

### Methodological quality of included studies

Quality assessment based on QUADAS guidelines was conducted on all 100 studies included for systematic review. Of the 100 eligible studies, 56 had a QUADAS score≥8, 21 had a QUADAS score = 7, 18 had a QUADAS score = 6, and five had a QUADAS score = 5. In the total included studies (see Figure S1 in file S1), greater than 90% of the included studies had high quality in terms of avoided partial verification, avoided differential verification, relevant clinical information and withdrawals explained. About 75%, 60% and 50% of the 100 studies had high quality in evaluation items in comparison to uninterpretable results reported(intermediate test results, incorporation avoided and the acceptable reference standard, respectively). Approximately 80% of the eligible studies were unclear about whether the index test results were blinded (whether the investigators that determined the index test results were blind to the patient?). Regarding the representative spectrum, approximately, 50% of the included studies had low quality, 40% were high quality and 10% did not report the information on how the participants were enrolled.

### Diagnostic accuracy

In 100 eligible studies with 23 different types of cancers, there were 13 types of cancers that could be subjected to meta-analysis (Table 1). Meta-analysis could not be conducted on seven types of cancers (vulvar, brain, gestational trophoblastic, soft connective tissue, skin, urethral, and genito-urinary tumors) because each tumor type had only one study included. We did not conduct meta-analysis on the remaining three (chronic myelogenous leukemia, nasopharynx cancer, thyroid cancer) types of cancer because the primary studies did not focus on the single cancer, but rather on varieties of cancers, and did not report detailed information about the methodological quality of the studies. For all of the cancers included in the 100 studies, the pooled DOR was 7.56 (95%CI: 6.02–9.50), indicating that s-p53 antibody could be a useful biomarker for cancer patient diagnosis. There appeared to be qualitative evidence for heterogeneity between studies (I^2^ = 48.9%). We analysed the symmetrical SROC of s-p53 antibody and the AUC was 0.67, indicating that s-p53 antibodies had reasonable accuracy in terms of differential diagnosis in cases of cancer. Of the 100 eligible studies, sensitivity and specificity ranged from 2.90%–68.30% and 67.30%–100%, respectively. In the present study, a pooled PLR of 5.75 (95% CI: 4.60–7.19) suggests that patients with cancer have approximately 6-fold higher chance of being s-p53 antibody-positive compared with patients without cancer. Also, there was heterogeneity between PLRs, with I^2^ = 48.90%. Similarly, we found significant heterogeneity for all of the eligible studies regarding NLR, with I^2^ = 91.10%. The pooled negative likelihood ratio was 0.81 (95% CI: 0.79–0.83), indicating that patients without cancer have a 1.25-fold higher chance of being s-p53 antibody-negative compared with patients with cancer. Therefore, being positive for s-p53 antibody had more diagnostic value than being negative in clinical practice for detecting cancer.

**Table 1:** Pooled diagnostic accuracy of s-p53 antibody for detection of 13 types of cancer.

Tumor	No.	Case	Control	PLR	NLR	DOR	AUC
		(n)	(n)	(95%CI)*	(95%CI)*	(95%CI)*	
Bladder	5	189	1508	5.24	0.88	6.01	0.29
				(1.50–18.35)	(0.82–0.95)	(1.65–21.89)	
Breast	12	1739	2777	9.09	0.85	10.85	0.71
				(4.92–16.78)	(0.80–0.90)	(5.84–20.16)	
Colorectal	16	2781	3367	11.05	0.80	13.80	0.67
				(6.16–19.82)	(0.75–0.84)	(8.15–23.36)	
Esophagus	15	1079	2295	6.95	0.75	9.65	0.74
				(4.77–9.51)	(0.72–0.78)	(7.04–13.22)	
Gastric	6	882	185	10.82	0.83	13.64	0.70
				(3.49–33.55)	(0.77–0.89)	(4.25–43.78)	
Head and	7	461	1522	4.61	0.79	5.63	0.40
neck				(2.24–9.50)	(0.72–0.88)	(2.88–11.02)	
hepatic	17	1334	2718	4.36	0.84	5.65	0.75
				(2.23–8.53)	(0.76–0.92)	(2.74–11.66)	
Lung	21	2382	3001	6.61	0.86	7.80	0.59
				(3.92–11.16)	(0.82–0.90)	(4.58–13.31)	
Lymphoma	7	487	1301	4.42	0.86	6.79	0.81
				(2.48–7.88)	(0.76–0.97)	(3.50–13.18)	
Oral	5	314	352	2.33	0.83	2.86	0.47
				(1.43–3.80)	(0.77–0.88)	(1.68–4.88)	
Ovarian	11	4572	2056	6.13	0.86	7.45	0.65
				(3.83–9.81)	(0.80–0.91)	(4.69–11.84)	
Prostatic	4	179	1631	3.88	0.97	4.35	0.57
				(1.77–8.50)	(0.92–1.03)	(1.80–10.62)	
Pancreatic	10	437	2288	6.05	0.88	7.18	0.46
				(2.50–14.64)	(0.82–0.95)	(2.83–18.21)	
Total	136	16822	25015				

Note: PLR: positive likelihood ratio, NLR: negative likelihood ratio, DOR: diagnostic odds ratio, AUC: the area under the SROC curve; PLR (95% CI)*, DOR (95% CI)* and NLR (95% CI)* were calculated using a random effect model.

For meta-analysis for all 13 types of cancers, we used the same statistical analysis methods and indicators as above for the 100 eligible studies to individually evaluate the diagnostic accuracy of s-p53 antibody for a single cancer. As shown in Table 1, the ranges of the PLR, NLR, DOR, AUC, and positive rate were (2.33–11.05), (0.74–0.97), (2.86–13.8), (0.29–0.81), (4.47%–28.36%), respectively. We found that breast, colorectal, esophageal, gastric, hepatic, lymphoma, lung and ovarian cancer had a relatively reasonable diagnostic accuracy. The remaining pooled results of the five types of cancers suggested that s-p53 antibody had limited value for diagnosis, especially for oral cancer. Additionally, we listed the rang of the sensitivity and specificity for 13 different types of the cancer (see Figure S2–S14 in file S1).

In our meta-analyses, there were 38 studies that included benign disease as a negative control. Pooled analysis results of the above 38 studies showed lower diagnostic accuracy than the pooled results of the 100 included studies. The results of the meta-analysis showed a PLR of 3.28 (95%CI: 2.32–4.62), NLR of 0.83 (95%CI: 0.80–0.87), DOR of 4.28 (95%CI: 2.93–6.26), and AUC of 0.58. This also indicated that restricted design of the study could produce more objective results, which generally tended to be on the verge of suitability for clinical practice. Furthermore, the comparison between the cancer and corresponding benign disease objectively indicated that s-p53 antibody had potential diagnostic value for cancer.

### Possible sources of heterogeneity

The threshold for calling a result indeterminate may differ between studies. Computation of the Spearman correction coefficient between the logit of sensitivity and logit of 1-specificity of s-p53 antibody was 0.322 (P = 0.001), indicating there was a threshold effect, and the positive correlation had statistical significance [21]. Meta-regression and sub-group analyses were used to explore the overall heterogeneity and possible sources of heterogeneity, which may include variation in quality of methodology in the studies (QUADAS), assay method, representation of participants (the percentage of the stage I in cancers), negative controls, and/or sample collection times between each study. Meta-regression indicated that the above variables were not the sources of heterogeneity for s-p53-antibody because all of the p values were larger than 0.05. The RDOR(relative diagnostic odds ratio)value was more than one regarding blinding, standard reference, and negative control (data not shown). If possible, we conducted subgroup analysis for all of the covariants we extracted (Table 2). The 100 eligible studies were stratified by overall score into 2 groups: scores≥8 (n = 56) and scores<8 (n = 44). There was a difference between the performance of data sets that scored≥8 (DOR, 5.92) compared to scores of <8 (DOR, 10.28). Studies were grouped based on their assay method [ELISA (n = 85) or others (n = 13)]. The ELISA assay method (DOR, 7.08) had lower diagnostic precision than the other assay methods (DOR, 12.04), such as immunoblot or western-blot. There was also a difference between the test performance of stage I%>20% (n = 15, DOR, 7.28) and stage I%≦20% (n = 26, DOR, 7.38). Three different types of negative control were used: healthy controls (n = 54), benign disease controls (n = 20), and healthy and benign disease controls (n = 20). The diagnostic accuracy of the three sub-groups were as follows: healthy control (DOR, 10.41), benign disease control (DOR, 4.20), and healthy and benign disease control (DOR, 7.02). However, there was no difference between the subgroup of the sample collection time [before treatment (n = 20), DOR, 7.25; before diagnosis (n = 7), DOR, 6.12]. From the subgroup analysis results above, the main heterogeneity sources were study quality (QUADAS), assay method, stage I%, and negative controls.

**Table 2:** Possible sources of heterogeneity of sub-group analysis.

Subgroup	(n)	PLR (95% CI)*	NLR (95% CI)*	DOR (95% CI)*
QUADAS	≥8(n = 56)	4.27 (3.29–5.56)	0.80 (0.77–0.83)	5.92 (4.42–7.92)
	<8 (n = 44)	7.96 (5.77–10.98)	0.82 (0.79– 0.85)	10.28 (7.40–14.28)
Assay method	ELISA (n = 85)	5.33 (4.25–6.68)	0.81 (0.79–0.83)	7.08 (5.59–8.96)
	Other (n = 13)	9.46 (4.25–21.06)	0.83 (0.75–0.92)	12.04 (5.33- 27.18)
Stage I%	>20% (n = 15)	5.84 (3.42–9.98)	0.81 (0.75–0.86)	7.28 (4.90–10.83)
	< = 20% (n = 26)	5.95 (3.74–9.47)	0.82 (0.78–0.87)	7.38 (4.703–11.57)
Negative control	Health (n = 54)	7.86 (5.98–10.33)	0.80 (0.78–0.83)	10.41 (7.86–13.80)
	benign disease (n = 20)	5.89 (2.98–11.64)	0.85 (0.81–0.89)	7.02 (3.48–14.14)
	Health+benign disease(n = 20)	2.89 (2.18–3.83)	0.77 (0.70–0.83)	4.20 (2.92–6.02)
Sample collection	Before treatment (n = 20)	8.76 (5.61–13.69)	0.82 (0.77–0.86)	11.90 (7.42–19.07)
	Before diagnosis (n = 7)	5.73 (2.67–12.29)	0.78 (0.68–0.89)	7.77 (3.81–15.88)
Study design	prospective (n = 3)	11.55 (3.71–35.97)	0.87 (0.83–0.90)	13.35 (4.21–42.38)
	retrospective(n = 97)	5.66 (4.52–7.08)	0.81 (0.78–0.83)	7.48 (5.93–9.43)

Note: QUADAS: quality assessment of studies of diagnostic accuracy, PLR: positive likelihood ratio, NLR: negative likelihood ratio, DOR: diagnostic odds ratio. PLR (95% CI)*, DOR (95% CI)* and NLR (95% CI)* were calculated using a random effect model.

### Sensitivity analysis and publication bias

**Figure 2:**
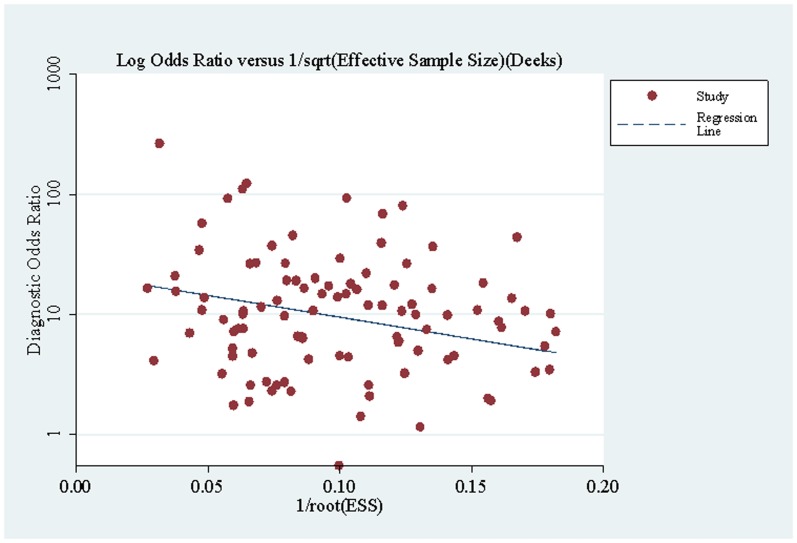
Funnel plot for the assessment of potential bias in s-p53 antibody assays. The funnel graph plots the DOR (diagnostic odds ratio) against the 1/root (effective sample size). The dotted line is the regression line. The result of the test for publication bias showed publication bias (p<0.001).

To determine whether any single data set was incurring undue weight in the analysis, we systematically removed 1 data set at a time and computed I^2^ for the remaining group. This was conducted for statistical analysis methods, study quality, sample size and study design. We used a fixed effect model to analyze the data again to replace the random effect model, but the results produced no obvious changes. When we excluded the studies (QUADAS score≦6, n = 23) to pooled the data(QUADAS score>6, n = 77), the results were as good as the results of the 100 eligible studies. When we excluded the studies (n = 15) without matched cases and control sample size, the results were similar to the original results. In addition, when we excluded the studies that studied various cancers (n = 9), but did not provide detailed information of the participants, the results remained unchanged, indicating that our meta-analysis provided stabilized results. A Deek's funnel plot(Figure 2) showed an asymmetric distribution of the points in the funnel plot for detection of publication bias (intercept, 3.09; 95%CI,2.54–3.63; P = 0.000), indicating that publication bias was likely.

## Discussion

In a systematic review of the published literature, we find that patients with cancer have a higher chance of being s-p53 antibody-positive compared with patients without cancer, and that the ratio of the odds of a positive test result among cancer patients is approximately 6-fold the odds of a positive test result among non-cancer individuals. Furthermore, the ratio of the odds of a positive test result among cancer patients is approximately 3-fold the odds of a positive test result for benign disease. In brief, the positive frequency of s-p53 antibody in most of the cancer patients is higher than in healthy and benign controls. Therefore, a positive s-p53 antibody test is diagnostic of cancer. This results are line with the published article[22], which is the part of the this article.

It is not uncommon that s-p53 antibody could be detected in most cancers. Studies of the molecular biology of malignant tumors emphasize the importance of a number of proto-oncogenes and tumor suppressor genes in human malignancies. Thus, the search for biomarkers that can diagnose various types of malignancies is important for the better management of patients. Several studies report that serum p53 antibodies (s-p53 Abs) are detected in different populations that are at increased risk for developing malignant disease [23], [24]. Positive rates for markers in cancer might be different according to the disease stage of the patient because anti-p53 may accumulate in the early stages of carcinogenesis. Shigeo Yoshizawa thought p53 Abs are usually IgG, indicating a secondary response after prolonged immunization by p53 protein accumulation; thus it is reasonable to presume that such p53 Abs could be used as an early indicator of p53 mutations in tumors in which such alterations occur early during tumoral progression(see Reference 89 in File S1). In a previous study(see Reference 57 in File S1), positive rates for anti-p53 in clinical cancer stages I and II ranged from 33% to 50% and were greater than those found in stages III and IV. S-p53 Abs can be used to follow the response of patients with malignant tumors during treatment (see Reference 22 in File S1). In addition, this strategy of combining markers could be a plausible approach to the low-positivity issue of conventional markers in screening for lung cancer. Actually, Yongjung Park(see Reference 99 in File S1) found that the combination of 2 or 3 markers, including anti-p53, had greater AUC values than did a single marker or the combination of other markers without anti-p53. Because the ELISA assay is a quick and convenient assay for detecting p53 antibodies, s-p53 Abs may serve as a useful marker for diagnosis in cancer patient groups. According to our meta-analysis, we provide evidence that detection of s-p53 antibody is potentially valuable for cancer diagnosis (AUC = 0.71). In 13 different types of tumors for which we conducted meta-analysis, there is distinct diagnostic value for s-p53 antibodies. Surprisingly, our meta-analysis results show that for lymphoma, esophageal, hepatic, colorectal, gastric, ovarian, and lung cancer, s-p53 Abs have reasonable diagnostic value (Table 1). The AUC value, which is indicative of diagnostic ability, was as follows: lymphoma (0.81), esophageal (0.74), hepatic (0.75), breast (0.71), colorectal (0.67), gastric (0.70), ovarian (0.65), and lung (0.59) cancers. QUADAS, which can be used for systematic review of diagnostic accuracy studies, was used to evaluate the methodological quality of the included studies. Our meta-analysis shows that the methodological quality of reports on diagnostic research of s-p53-antibody is moderate, as determined by the QUADAS tool [25].

In meta-analysis, pooled indicators are usually used for homogeneity studies. However, most diagnostic reviews show considerable heterogeneity between the included studies because of the different cut-off values and assay methods [8]. It is very important to note that the point estimates of PLR, NLR and DOR must be carefully evaluated, and the sources of heterogeneity between studies should be searched and explained. In studies presented here, the Spearman correlation coefficient indicates that a threshold effect is the source of heterogeneity because different studies had different cut-off values. Furthermore, the validation assay for s-p53 antibody used in each study was different; some used ELISA, whereas others used immunoblotting or both, adding additional heterogeneity. Studies involving healthy controls tend to show higher specificity, than those recruiting patients with clinically suspected disease, consecutively and prospectively in a representative clinical setting. Therefore, the distinct type of negative control may also be a main source of heterogeneity. The distinct percentage of the patients being stage I among the 100 eligible studies may also lead to significant heterogeneity. Although our meta-regression did not show the source of heterogeneity between studies, subgroup analysis suggests a difference between the different subgroups.

Although we tried to avoid bias in the process of identifying studies, screening, assessing, data extraction, and data analyses, the present study has several limitations. First, we did not calculate the diagnostic accuracy for the early stage (stage I-II) cancers because sufficient raw data was not provided. Although we aimed to evaluate the diagnostic value of s-p53 antibodies for the early diagnosis of the cancer, cancer patients regardless of disease stage were used to evaluate the diagnostic power because of the limitation of information. Primary data were unavailable for investigation of elevated or decreased s-p53 antibody values as a function of tumor type, histology, age, or degree. Second, 54 of the 100 included studies used healthy controls, whereas only 20 studies used benign disease, and 20 studies used both healthy controls and benign controls, which strongly exaggerated the diagnostic accuracy. A higher value of DOR is obtained between cancer patients and health controls (DOR, 10.41, 95%CI: 7.86–13.80) than between cancer patients and benign disease (DOR, 4.20, 95%CI: 2.92–6.02). Although the non-restricted design could overestimate the discrimination power of s-p53-antibodies in cancer, the meta-analysis based on comprehensive, large sample quantitative assessments provides more convincing evidence. Indeed, evidence for the diagnostic value of s-p53 antibody for cancer is compelling in that the PLR, DOR values were all larger than five. Thirdly, systematic reviewers are advised to use comprehensive searches to attempt to locate all relevant studies [26]–[28]. However, we observed significant publication bias in our study. We did not attempt to quantify the number of unpublished studies but realize that conclusion may be too optimistic when studies with favorable results are more likely to be submitted and published. It has been reported that exclusion of unpublished studies can yield a 15% larger intervention effect, but such data are not available for diagnostic research on bias between studies because we missed the unpublished studies [8]. Finally, we excluded 20 studies because they did not provide data allowing construction of 2×2 tables. We did not contact authors to obtain further data, potentially resulting in biased results and less precise estimates of pooled diagnostic accuracy.

In conclusion, the current evidence suggests that s-p53 antibody is a useful biomarker for cancer diagnosis, especially for breast, colorectal, esophageal, gastric, hepatic, lymphoma, lung and ovarian cancers. Significantly, there few individuals in healthy controls display s-p53 antibodies. However, it is not uncommon that the frequency of s-p53 antibody-positive individuals is different between most types of cancer patients and healthy controls. Patients with cancer have a higher chance of being s-p53 antibody-positive compared with patients without cancer.

## Supporting Information

File S1 **Supporting figures, tables and references.** Figure S1 Methodological quality graph: review authors' judgments about each methodological quality item presented as percentages across all included studies. (PDF). Figure S2 Forest plot of sensitivity and specificity of 5 individual studies for s-p53-antibody in the diagnosis of bladder cancer. The point estimates of sensitivity/specificity from each study are shown as solid circles. Error bars are 95% confidence intervals. Figure S3 Forest plot of sensitivity and specificity of 12 individual studies for s-p53-antibody in the diagnosis of breast cancer. The point estimates of sensitivity/specificity from each study are shown as solid circles. Error bars are 95% confidence intervals. Figure S4 Forest plot of sensitivity and specificity of 16 individual studies for s-p53-antibody in the diagnosis of colorectal cancer. The point estimates of sensitivity/specificity from each study are shown as solid circles. Error bars are 95% confidence intervals. Figure S5 Forest plot of sensitivity and specificity of 15 individual studies for s-p53-antibody in the diagnosis of EC. The point estimates of sensitivity/specificity from each study are shown as solid circles. Error bars are 95% confidence intervals. Figure S6 Forest plot of sensitivity and specificity of 6 individual studies for s-p53-antibody in the diagnosis of gastric cancer. The point estimates of sensitivity/specificity from each study are shown as solid circles. Error bars are 95% confidence intervals. Figure S7 Forest plot of sensitivity and specificity of 7 individual studies for s-p53-antibody in the diagnosis of head and neck cancer. The point estimates of sensitivity/specificity from each study are shown as solid circles. Error bars are 95% confidence intervals. Figure S8 Forest plot of sensitivity and specificity of 17 individual studies for s-p53-antibody in the diagnosis of hepatocellular carcinoma. The point estimates of sensitivity/specificity from each study are shown as solid circles. Error bars are 95% confidence intervals. Figure S9 Forest plot of sensitivity and specificity of 21 individual studies for s-p53-antibody in the diagnosis of lung cancer. The point estimates of sensitivity/specificity from each study are shown as solid circles. Error bars are 95% confidence intervals. Figure S10 Forest plot of sensitivity and specificity of 7 individual studies for s-p53-antibody in the diagnosis of lymph cancer. The point estimates of sensitivity /specificity from each study are shown as solid circles. Error bars are 95% confidence intervals. Figure S11 Forest plot of sensitivity and specificity of 5 individual studies for s-p53-antibody in the diagnosis of oral cancer. The point estimates of sensitivity/specificity from each study are shown as solid circles. Error bars are 95% confidence intervals. Figure S12 Forest plot of sensitivity and specificity of 11 individual studies for s-p53-antibody in the diagnosis of ovarian cancer. The point estimates of sensitivity/specificity from each study are shown as solid circles. Error bars are 95% confidence intervals. Figure S13 Forest plot of sensitivity and specificity of 4 individual studies for s-p53-antibody in the diagnosis of prostate cancer. The point estimates of sensitivity/specificity from each study are shown as solid circles. Error bars are 95% confidence intervals. Figure S14 Forest plot of sensitivity and specificity of 10 individual studies for s-p53-antibody in the diagnosis of pancreatic cancer. The point estimates of sensitivity/specificity from each study are shown as solid circles. Error bars are 95% confidence intervals. Table S1 Main characteristics of the 100 eligible studies (a, b). Table S2 Search strategy in PubMed. (PDF). Additional file: Reference (Included studies).
(ZIP)

Checklist S1 **PRISMA Checklist.**
(DOC)
