# Supplementary figures and images for: Assessment of the Potential Diagnostic Value of Serum p53 Antibody for Cancer: A Meta-Analysis
Source: PLoS One. 2014 Jun 9;9(6):e99255. doi: 10.1371/journal.pone.0099255 (PMC4049633; doi:10.1371/journal.pone.0099255)

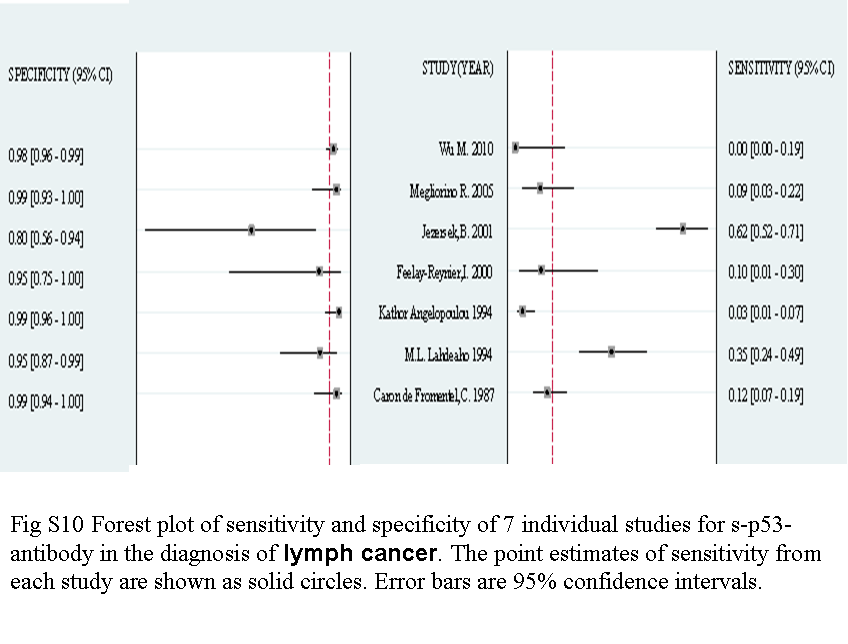

Supplement: File S1 — Supporting figures, tables and references. Figure S1 Methodological quality graph: review authors' judgments about each methodological quality item presented as percentages across all included studies. (PDF). Figure S2 Forest plot of sensitivity and specificity of 5 individual studies for s-p53-antibody in the diagnosis of bladder cancer. The point estimates of sensitivity/specificity from each study are shown as solid circles. Error bars are 95% confidence intervals. Figure S3 Forest plot of sensitivity and specificity of 12 individual studies for s-p53-antibody in the diagnosis of breast cancer. The point estimates of sensitivity/specificity from each study are shown as solid circles. Error bars are 95% confidence intervals. Figure S4 Forest plot of sensitivity and specificity of 16 individual studies for s-p53-antibody in the diagnosis of colorectal cancer. The point estimates of sensitivity/specificity from each study are shown as solid circles. Error bars are 95% confidence intervals. Figure S5 Forest plot of sensitivity and specificity of 15 individual studies for s-p53-antibody in the diagnosis of EC. The point estimates of sensitivity/specificity from each study are shown as solid circles. Error bars are 95% confidence intervals. Figure S6 Forest plot of sensitivity and specificity of 6 individual studies for s-p53-antibody in the diagnosis of gastric cancer. The point estimates of sensitivity/specificity from each study are shown as solid circles. Error bars are 95% confidence intervals. Figure S7 Forest plot of sensitivity and specificity of 7 individual studies for s-p53-antibody in the diagnosis of head and neck cancer. The point estimates of sensitivity/specificity from each study are shown as solid circles. Error bars are 95% confidence intervals. Figure S8 Forest plot of sensitivity and specificity of 17 individual studies for s-p53-antibody in the diagnosis of hepatocellular carcinoma. The point estimates of sensitivity/specificity from each study ar [file pone.0099255.s001.zip › supplementary materials--Dec/Fig S10.bmp]

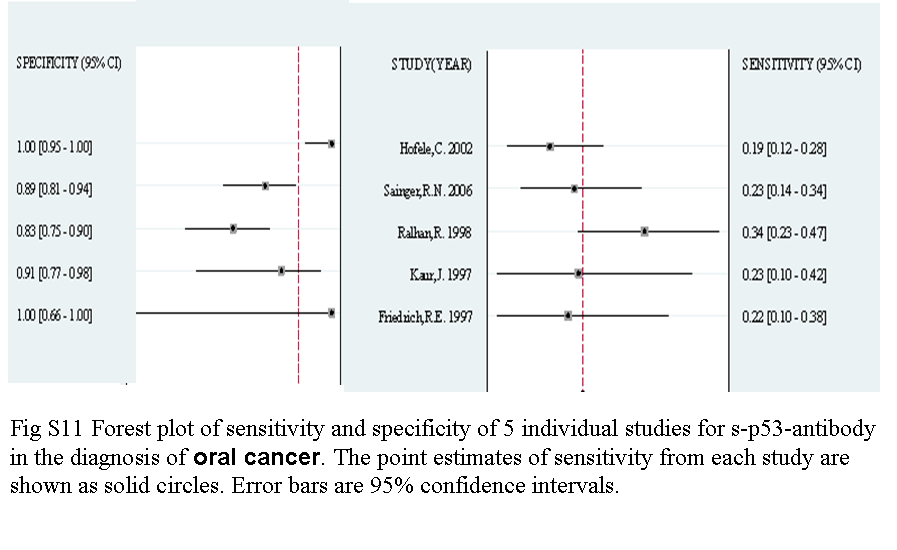

Supplement: File S1 — Supporting figures, tables and references. Figure S1 Methodological quality graph: review authors' judgments about each methodological quality item presented as percentages across all included studies. (PDF). Figure S2 Forest plot of sensitivity and specificity of 5 individual studies for s-p53-antibody in the diagnosis of bladder cancer. The point estimates of sensitivity/specificity from each study are shown as solid circles. Error bars are 95% confidence intervals. Figure S3 Forest plot of sensitivity and specificity of 12 individual studies for s-p53-antibody in the diagnosis of breast cancer. The point estimates of sensitivity/specificity from each study are shown as solid circles. Error bars are 95% confidence intervals. Figure S4 Forest plot of sensitivity and specificity of 16 individual studies for s-p53-antibody in the diagnosis of colorectal cancer. The point estimates of sensitivity/specificity from each study are shown as solid circles. Error bars are 95% confidence intervals. Figure S5 Forest plot of sensitivity and specificity of 15 individual studies for s-p53-antibody in the diagnosis of EC. The point estimates of sensitivity/specificity from each study are shown as solid circles. Error bars are 95% confidence intervals. Figure S6 Forest plot of sensitivity and specificity of 6 individual studies for s-p53-antibody in the diagnosis of gastric cancer. The point estimates of sensitivity/specificity from each study are shown as solid circles. Error bars are 95% confidence intervals. Figure S7 Forest plot of sensitivity and specificity of 7 individual studies for s-p53-antibody in the diagnosis of head and neck cancer. The point estimates of sensitivity/specificity from each study are shown as solid circles. Error bars are 95% confidence intervals. Figure S8 Forest plot of sensitivity and specificity of 17 individual studies for s-p53-antibody in the diagnosis of hepatocellular carcinoma. The point estimates of sensitivity/specificity from each study ar [file pone.0099255.s001.zip › supplementary materials--Dec/Fig S11.bmp]

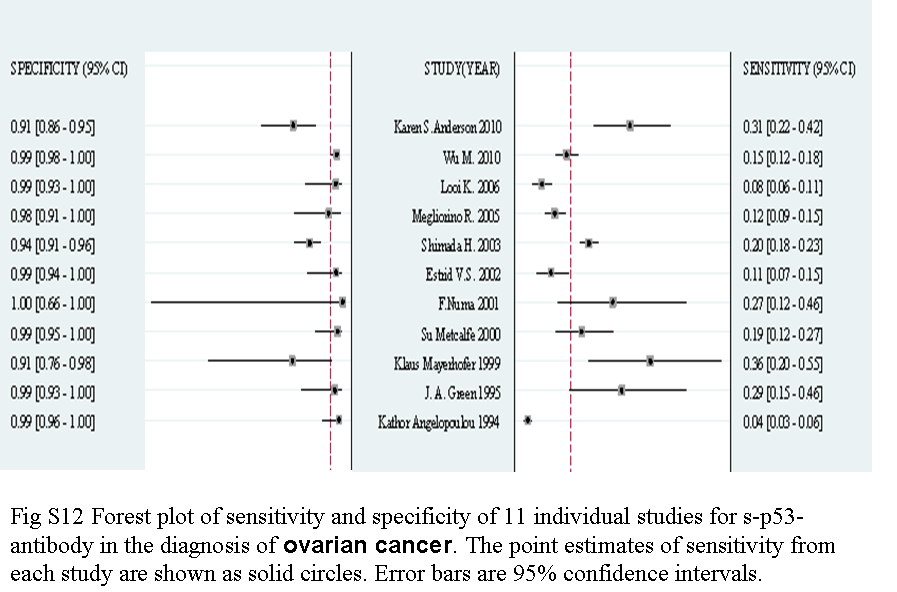

Supplement: File S1 — Supporting figures, tables and references. Figure S1 Methodological quality graph: review authors' judgments about each methodological quality item presented as percentages across all included studies. (PDF). Figure S2 Forest plot of sensitivity and specificity of 5 individual studies for s-p53-antibody in the diagnosis of bladder cancer. The point estimates of sensitivity/specificity from each study are shown as solid circles. Error bars are 95% confidence intervals. Figure S3 Forest plot of sensitivity and specificity of 12 individual studies for s-p53-antibody in the diagnosis of breast cancer. The point estimates of sensitivity/specificity from each study are shown as solid circles. Error bars are 95% confidence intervals. Figure S4 Forest plot of sensitivity and specificity of 16 individual studies for s-p53-antibody in the diagnosis of colorectal cancer. The point estimates of sensitivity/specificity from each study are shown as solid circles. Error bars are 95% confidence intervals. Figure S5 Forest plot of sensitivity and specificity of 15 individual studies for s-p53-antibody in the diagnosis of EC. The point estimates of sensitivity/specificity from each study are shown as solid circles. Error bars are 95% confidence intervals. Figure S6 Forest plot of sensitivity and specificity of 6 individual studies for s-p53-antibody in the diagnosis of gastric cancer. The point estimates of sensitivity/specificity from each study are shown as solid circles. Error bars are 95% confidence intervals. Figure S7 Forest plot of sensitivity and specificity of 7 individual studies for s-p53-antibody in the diagnosis of head and neck cancer. The point estimates of sensitivity/specificity from each study are shown as solid circles. Error bars are 95% confidence intervals. Figure S8 Forest plot of sensitivity and specificity of 17 individual studies for s-p53-antibody in the diagnosis of hepatocellular carcinoma. The point estimates of sensitivity/specificity from each study ar [file pone.0099255.s001.zip › supplementary materials--Dec/Fig S12.bmp]

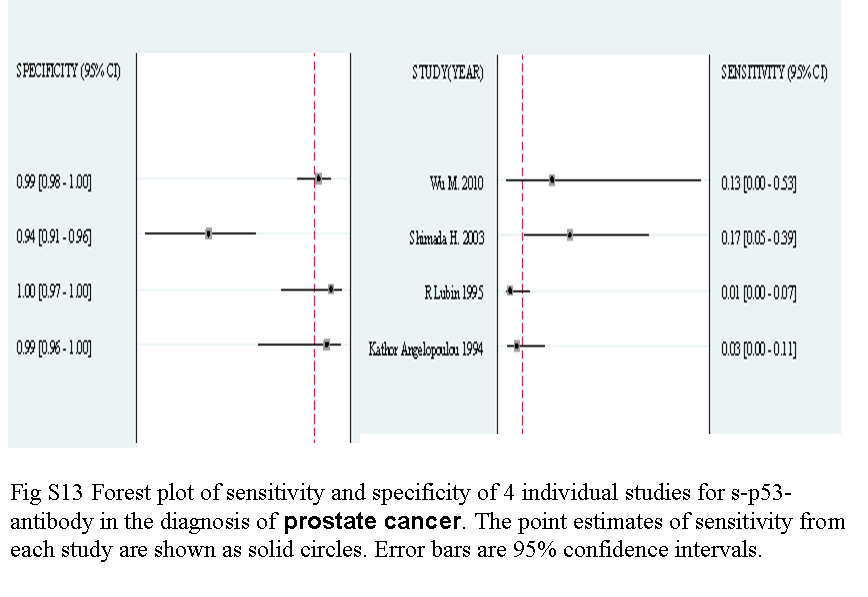

Supplement: File S1 — Supporting figures, tables and references. Figure S1 Methodological quality graph: review authors' judgments about each methodological quality item presented as percentages across all included studies. (PDF). Figure S2 Forest plot of sensitivity and specificity of 5 individual studies for s-p53-antibody in the diagnosis of bladder cancer. The point estimates of sensitivity/specificity from each study are shown as solid circles. Error bars are 95% confidence intervals. Figure S3 Forest plot of sensitivity and specificity of 12 individual studies for s-p53-antibody in the diagnosis of breast cancer. The point estimates of sensitivity/specificity from each study are shown as solid circles. Error bars are 95% confidence intervals. Figure S4 Forest plot of sensitivity and specificity of 16 individual studies for s-p53-antibody in the diagnosis of colorectal cancer. The point estimates of sensitivity/specificity from each study are shown as solid circles. Error bars are 95% confidence intervals. Figure S5 Forest plot of sensitivity and specificity of 15 individual studies for s-p53-antibody in the diagnosis of EC. The point estimates of sensitivity/specificity from each study are shown as solid circles. Error bars are 95% confidence intervals. Figure S6 Forest plot of sensitivity and specificity of 6 individual studies for s-p53-antibody in the diagnosis of gastric cancer. The point estimates of sensitivity/specificity from each study are shown as solid circles. Error bars are 95% confidence intervals. Figure S7 Forest plot of sensitivity and specificity of 7 individual studies for s-p53-antibody in the diagnosis of head and neck cancer. The point estimates of sensitivity/specificity from each study are shown as solid circles. Error bars are 95% confidence intervals. Figure S8 Forest plot of sensitivity and specificity of 17 individual studies for s-p53-antibody in the diagnosis of hepatocellular carcinoma. The point estimates of sensitivity/specificity from each study ar [file pone.0099255.s001.zip › supplementary materials--Dec/Fig S13.bmp]

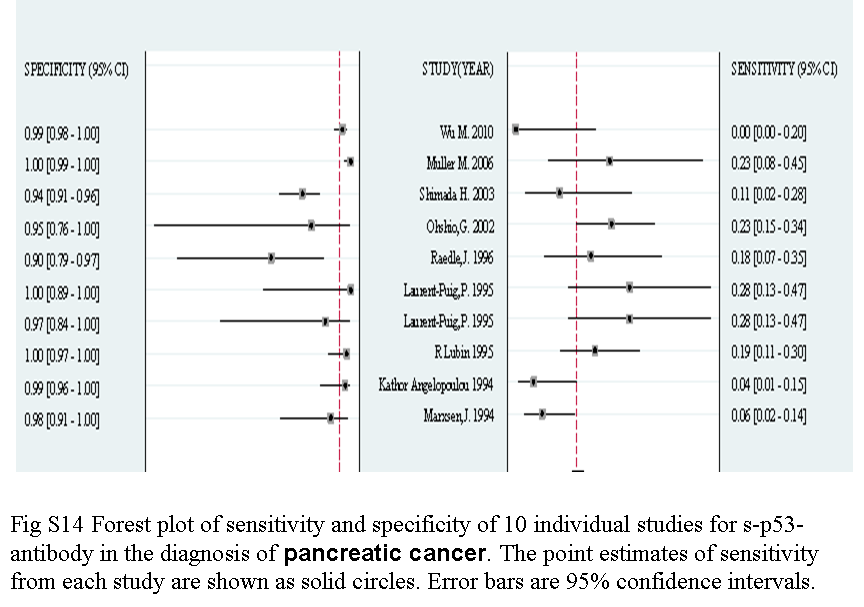

Supplement: File S1 — Supporting figures, tables and references. Figure S1 Methodological quality graph: review authors' judgments about each methodological quality item presented as percentages across all included studies. (PDF). Figure S2 Forest plot of sensitivity and specificity of 5 individual studies for s-p53-antibody in the diagnosis of bladder cancer. The point estimates of sensitivity/specificity from each study are shown as solid circles. Error bars are 95% confidence intervals. Figure S3 Forest plot of sensitivity and specificity of 12 individual studies for s-p53-antibody in the diagnosis of breast cancer. The point estimates of sensitivity/specificity from each study are shown as solid circles. Error bars are 95% confidence intervals. Figure S4 Forest plot of sensitivity and specificity of 16 individual studies for s-p53-antibody in the diagnosis of colorectal cancer. The point estimates of sensitivity/specificity from each study are shown as solid circles. Error bars are 95% confidence intervals. Figure S5 Forest plot of sensitivity and specificity of 15 individual studies for s-p53-antibody in the diagnosis of EC. The point estimates of sensitivity/specificity from each study are shown as solid circles. Error bars are 95% confidence intervals. Figure S6 Forest plot of sensitivity and specificity of 6 individual studies for s-p53-antibody in the diagnosis of gastric cancer. The point estimates of sensitivity/specificity from each study are shown as solid circles. Error bars are 95% confidence intervals. Figure S7 Forest plot of sensitivity and specificity of 7 individual studies for s-p53-antibody in the diagnosis of head and neck cancer. The point estimates of sensitivity/specificity from each study are shown as solid circles. Error bars are 95% confidence intervals. Figure S8 Forest plot of sensitivity and specificity of 17 individual studies for s-p53-antibody in the diagnosis of hepatocellular carcinoma. The point estimates of sensitivity/specificity from each study ar [file pone.0099255.s001.zip › supplementary materials--Dec/Fig S14.bmp]

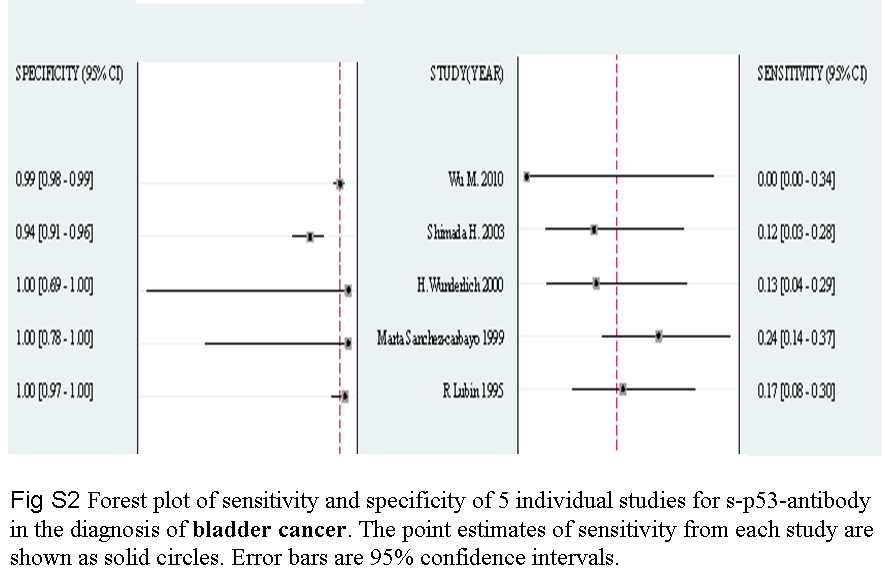

Supplement: File S1 — Supporting figures, tables and references. Figure S1 Methodological quality graph: review authors' judgments about each methodological quality item presented as percentages across all included studies. (PDF). Figure S2 Forest plot of sensitivity and specificity of 5 individual studies for s-p53-antibody in the diagnosis of bladder cancer. The point estimates of sensitivity/specificity from each study are shown as solid circles. Error bars are 95% confidence intervals. Figure S3 Forest plot of sensitivity and specificity of 12 individual studies for s-p53-antibody in the diagnosis of breast cancer. The point estimates of sensitivity/specificity from each study are shown as solid circles. Error bars are 95% confidence intervals. Figure S4 Forest plot of sensitivity and specificity of 16 individual studies for s-p53-antibody in the diagnosis of colorectal cancer. The point estimates of sensitivity/specificity from each study are shown as solid circles. Error bars are 95% confidence intervals. Figure S5 Forest plot of sensitivity and specificity of 15 individual studies for s-p53-antibody in the diagnosis of EC. The point estimates of sensitivity/specificity from each study are shown as solid circles. Error bars are 95% confidence intervals. Figure S6 Forest plot of sensitivity and specificity of 6 individual studies for s-p53-antibody in the diagnosis of gastric cancer. The point estimates of sensitivity/specificity from each study are shown as solid circles. Error bars are 95% confidence intervals. Figure S7 Forest plot of sensitivity and specificity of 7 individual studies for s-p53-antibody in the diagnosis of head and neck cancer. The point estimates of sensitivity/specificity from each study are shown as solid circles. Error bars are 95% confidence intervals. Figure S8 Forest plot of sensitivity and specificity of 17 individual studies for s-p53-antibody in the diagnosis of hepatocellular carcinoma. The point estimates of sensitivity/specificity from each study ar [file pone.0099255.s001.zip › supplementary materials--Dec/Fig S2.bmp]

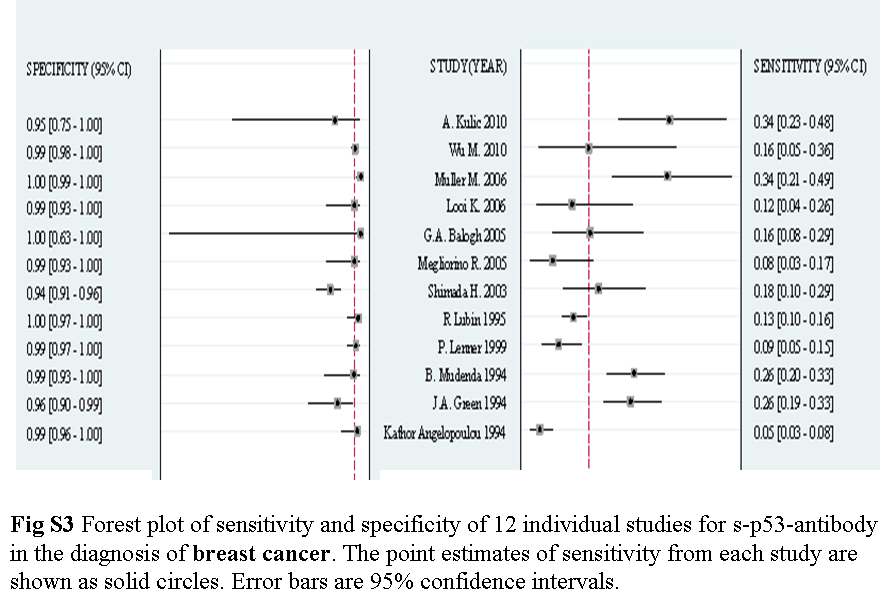

Supplement: File S1 — Supporting figures, tables and references. Figure S1 Methodological quality graph: review authors' judgments about each methodological quality item presented as percentages across all included studies. (PDF). Figure S2 Forest plot of sensitivity and specificity of 5 individual studies for s-p53-antibody in the diagnosis of bladder cancer. The point estimates of sensitivity/specificity from each study are shown as solid circles. Error bars are 95% confidence intervals. Figure S3 Forest plot of sensitivity and specificity of 12 individual studies for s-p53-antibody in the diagnosis of breast cancer. The point estimates of sensitivity/specificity from each study are shown as solid circles. Error bars are 95% confidence intervals. Figure S4 Forest plot of sensitivity and specificity of 16 individual studies for s-p53-antibody in the diagnosis of colorectal cancer. The point estimates of sensitivity/specificity from each study are shown as solid circles. Error bars are 95% confidence intervals. Figure S5 Forest plot of sensitivity and specificity of 15 individual studies for s-p53-antibody in the diagnosis of EC. The point estimates of sensitivity/specificity from each study are shown as solid circles. Error bars are 95% confidence intervals. Figure S6 Forest plot of sensitivity and specificity of 6 individual studies for s-p53-antibody in the diagnosis of gastric cancer. The point estimates of sensitivity/specificity from each study are shown as solid circles. Error bars are 95% confidence intervals. Figure S7 Forest plot of sensitivity and specificity of 7 individual studies for s-p53-antibody in the diagnosis of head and neck cancer. The point estimates of sensitivity/specificity from each study are shown as solid circles. Error bars are 95% confidence intervals. Figure S8 Forest plot of sensitivity and specificity of 17 individual studies for s-p53-antibody in the diagnosis of hepatocellular carcinoma. The point estimates of sensitivity/specificity from each study ar [file pone.0099255.s001.zip › supplementary materials--Dec/Fig S3.bmp]

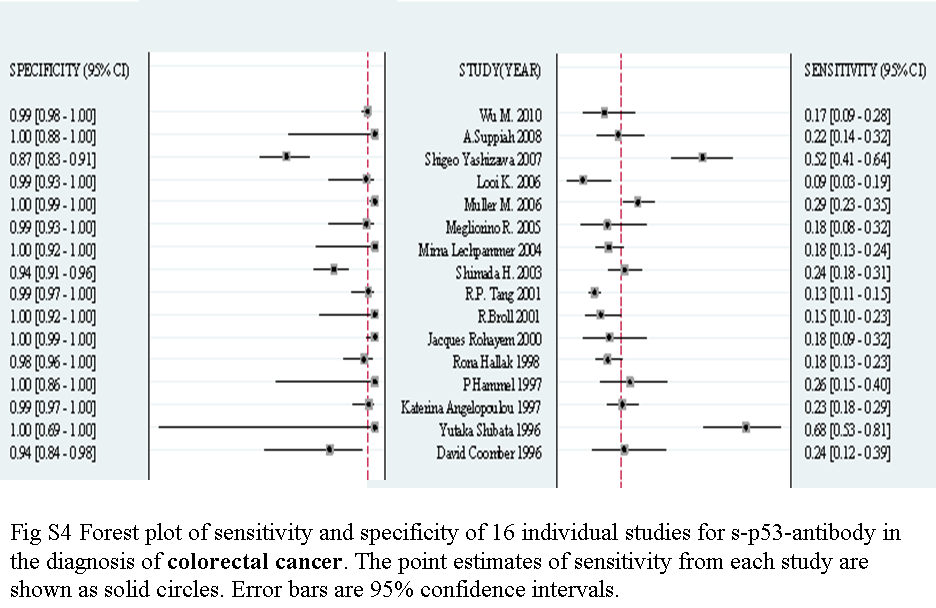

Supplement: File S1 — Supporting figures, tables and references. Figure S1 Methodological quality graph: review authors' judgments about each methodological quality item presented as percentages across all included studies. (PDF). Figure S2 Forest plot of sensitivity and specificity of 5 individual studies for s-p53-antibody in the diagnosis of bladder cancer. The point estimates of sensitivity/specificity from each study are shown as solid circles. Error bars are 95% confidence intervals. Figure S3 Forest plot of sensitivity and specificity of 12 individual studies for s-p53-antibody in the diagnosis of breast cancer. The point estimates of sensitivity/specificity from each study are shown as solid circles. Error bars are 95% confidence intervals. Figure S4 Forest plot of sensitivity and specificity of 16 individual studies for s-p53-antibody in the diagnosis of colorectal cancer. The point estimates of sensitivity/specificity from each study are shown as solid circles. Error bars are 95% confidence intervals. Figure S5 Forest plot of sensitivity and specificity of 15 individual studies for s-p53-antibody in the diagnosis of EC. The point estimates of sensitivity/specificity from each study are shown as solid circles. Error bars are 95% confidence intervals. Figure S6 Forest plot of sensitivity and specificity of 6 individual studies for s-p53-antibody in the diagnosis of gastric cancer. The point estimates of sensitivity/specificity from each study are shown as solid circles. Error bars are 95% confidence intervals. Figure S7 Forest plot of sensitivity and specificity of 7 individual studies for s-p53-antibody in the diagnosis of head and neck cancer. The point estimates of sensitivity/specificity from each study are shown as solid circles. Error bars are 95% confidence intervals. Figure S8 Forest plot of sensitivity and specificity of 17 individual studies for s-p53-antibody in the diagnosis of hepatocellular carcinoma. The point estimates of sensitivity/specificity from each study ar [file pone.0099255.s001.zip › supplementary materials--Dec/Fig S4.bmp]

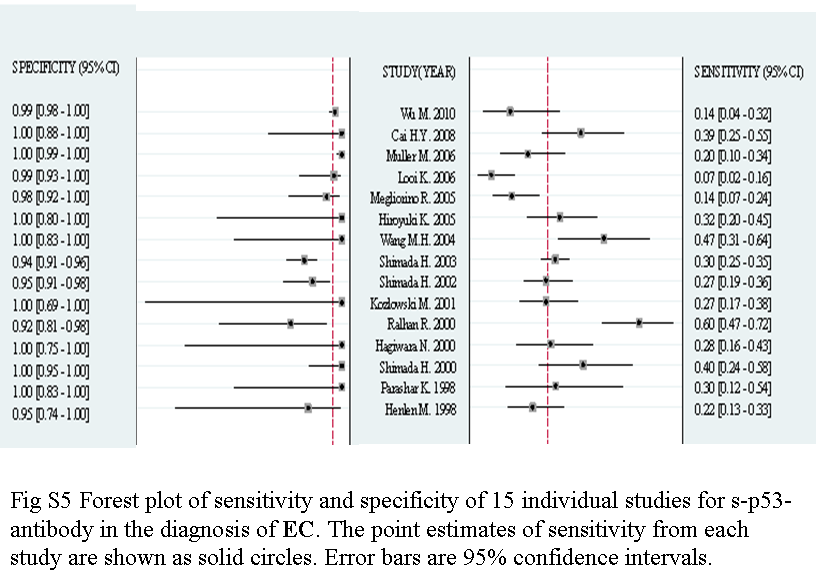

Supplement: File S1 — Supporting figures, tables and references. Figure S1 Methodological quality graph: review authors' judgments about each methodological quality item presented as percentages across all included studies. (PDF). Figure S2 Forest plot of sensitivity and specificity of 5 individual studies for s-p53-antibody in the diagnosis of bladder cancer. The point estimates of sensitivity/specificity from each study are shown as solid circles. Error bars are 95% confidence intervals. Figure S3 Forest plot of sensitivity and specificity of 12 individual studies for s-p53-antibody in the diagnosis of breast cancer. The point estimates of sensitivity/specificity from each study are shown as solid circles. Error bars are 95% confidence intervals. Figure S4 Forest plot of sensitivity and specificity of 16 individual studies for s-p53-antibody in the diagnosis of colorectal cancer. The point estimates of sensitivity/specificity from each study are shown as solid circles. Error bars are 95% confidence intervals. Figure S5 Forest plot of sensitivity and specificity of 15 individual studies for s-p53-antibody in the diagnosis of EC. The point estimates of sensitivity/specificity from each study are shown as solid circles. Error bars are 95% confidence intervals. Figure S6 Forest plot of sensitivity and specificity of 6 individual studies for s-p53-antibody in the diagnosis of gastric cancer. The point estimates of sensitivity/specificity from each study are shown as solid circles. Error bars are 95% confidence intervals. Figure S7 Forest plot of sensitivity and specificity of 7 individual studies for s-p53-antibody in the diagnosis of head and neck cancer. The point estimates of sensitivity/specificity from each study are shown as solid circles. Error bars are 95% confidence intervals. Figure S8 Forest plot of sensitivity and specificity of 17 individual studies for s-p53-antibody in the diagnosis of hepatocellular carcinoma. The point estimates of sensitivity/specificity from each study ar [file pone.0099255.s001.zip › supplementary materials--Dec/Fig S5.bmp]

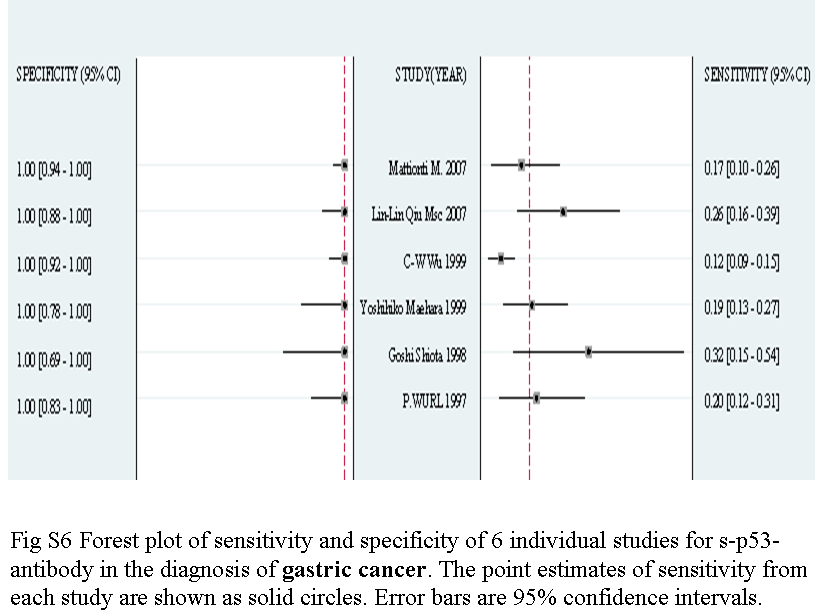

Supplement: File S1 — Supporting figures, tables and references. Figure S1 Methodological quality graph: review authors' judgments about each methodological quality item presented as percentages across all included studies. (PDF). Figure S2 Forest plot of sensitivity and specificity of 5 individual studies for s-p53-antibody in the diagnosis of bladder cancer. The point estimates of sensitivity/specificity from each study are shown as solid circles. Error bars are 95% confidence intervals. Figure S3 Forest plot of sensitivity and specificity of 12 individual studies for s-p53-antibody in the diagnosis of breast cancer. The point estimates of sensitivity/specificity from each study are shown as solid circles. Error bars are 95% confidence intervals. Figure S4 Forest plot of sensitivity and specificity of 16 individual studies for s-p53-antibody in the diagnosis of colorectal cancer. The point estimates of sensitivity/specificity from each study are shown as solid circles. Error bars are 95% confidence intervals. Figure S5 Forest plot of sensitivity and specificity of 15 individual studies for s-p53-antibody in the diagnosis of EC. The point estimates of sensitivity/specificity from each study are shown as solid circles. Error bars are 95% confidence intervals. Figure S6 Forest plot of sensitivity and specificity of 6 individual studies for s-p53-antibody in the diagnosis of gastric cancer. The point estimates of sensitivity/specificity from each study are shown as solid circles. Error bars are 95% confidence intervals. Figure S7 Forest plot of sensitivity and specificity of 7 individual studies for s-p53-antibody in the diagnosis of head and neck cancer. The point estimates of sensitivity/specificity from each study are shown as solid circles. Error bars are 95% confidence intervals. Figure S8 Forest plot of sensitivity and specificity of 17 individual studies for s-p53-antibody in the diagnosis of hepatocellular carcinoma. The point estimates of sensitivity/specificity from each study ar [file pone.0099255.s001.zip › supplementary materials--Dec/Fig S6.bmp]

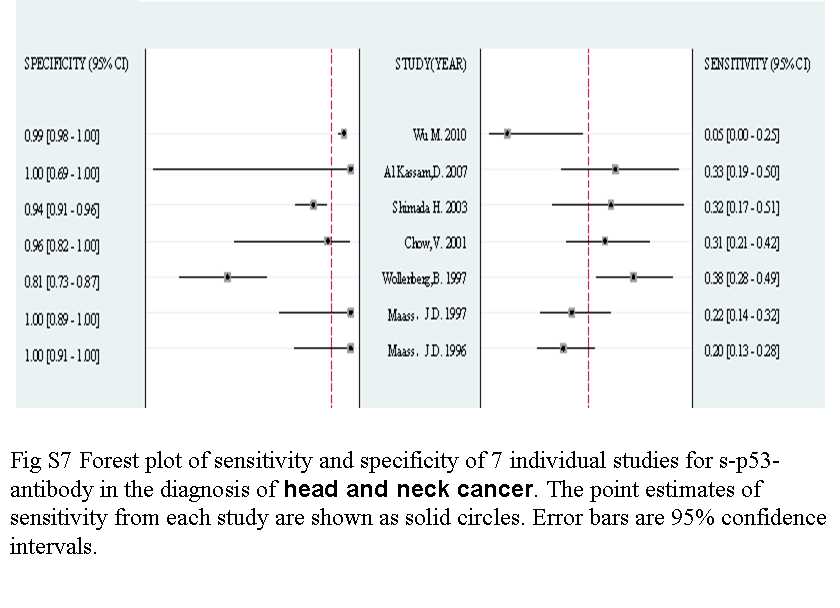

Supplement: File S1 — Supporting figures, tables and references. Figure S1 Methodological quality graph: review authors' judgments about each methodological quality item presented as percentages across all included studies. (PDF). Figure S2 Forest plot of sensitivity and specificity of 5 individual studies for s-p53-antibody in the diagnosis of bladder cancer. The point estimates of sensitivity/specificity from each study are shown as solid circles. Error bars are 95% confidence intervals. Figure S3 Forest plot of sensitivity and specificity of 12 individual studies for s-p53-antibody in the diagnosis of breast cancer. The point estimates of sensitivity/specificity from each study are shown as solid circles. Error bars are 95% confidence intervals. Figure S4 Forest plot of sensitivity and specificity of 16 individual studies for s-p53-antibody in the diagnosis of colorectal cancer. The point estimates of sensitivity/specificity from each study are shown as solid circles. Error bars are 95% confidence intervals. Figure S5 Forest plot of sensitivity and specificity of 15 individual studies for s-p53-antibody in the diagnosis of EC. The point estimates of sensitivity/specificity from each study are shown as solid circles. Error bars are 95% confidence intervals. Figure S6 Forest plot of sensitivity and specificity of 6 individual studies for s-p53-antibody in the diagnosis of gastric cancer. The point estimates of sensitivity/specificity from each study are shown as solid circles. Error bars are 95% confidence intervals. Figure S7 Forest plot of sensitivity and specificity of 7 individual studies for s-p53-antibody in the diagnosis of head and neck cancer. The point estimates of sensitivity/specificity from each study are shown as solid circles. Error bars are 95% confidence intervals. Figure S8 Forest plot of sensitivity and specificity of 17 individual studies for s-p53-antibody in the diagnosis of hepatocellular carcinoma. The point estimates of sensitivity/specificity from each study ar [file pone.0099255.s001.zip › supplementary materials--Dec/Fig S7.bmp]

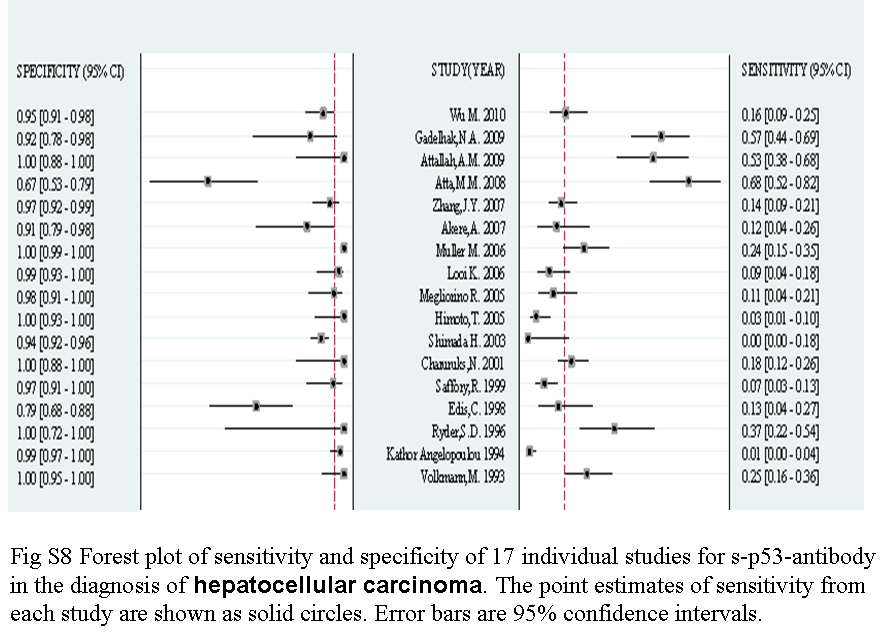

Supplement: File S1 — Supporting figures, tables and references. Figure S1 Methodological quality graph: review authors' judgments about each methodological quality item presented as percentages across all included studies. (PDF). Figure S2 Forest plot of sensitivity and specificity of 5 individual studies for s-p53-antibody in the diagnosis of bladder cancer. The point estimates of sensitivity/specificity from each study are shown as solid circles. Error bars are 95% confidence intervals. Figure S3 Forest plot of sensitivity and specificity of 12 individual studies for s-p53-antibody in the diagnosis of breast cancer. The point estimates of sensitivity/specificity from each study are shown as solid circles. Error bars are 95% confidence intervals. Figure S4 Forest plot of sensitivity and specificity of 16 individual studies for s-p53-antibody in the diagnosis of colorectal cancer. The point estimates of sensitivity/specificity from each study are shown as solid circles. Error bars are 95% confidence intervals. Figure S5 Forest plot of sensitivity and specificity of 15 individual studies for s-p53-antibody in the diagnosis of EC. The point estimates of sensitivity/specificity from each study are shown as solid circles. Error bars are 95% confidence intervals. Figure S6 Forest plot of sensitivity and specificity of 6 individual studies for s-p53-antibody in the diagnosis of gastric cancer. The point estimates of sensitivity/specificity from each study are shown as solid circles. Error bars are 95% confidence intervals. Figure S7 Forest plot of sensitivity and specificity of 7 individual studies for s-p53-antibody in the diagnosis of head and neck cancer. The point estimates of sensitivity/specificity from each study are shown as solid circles. Error bars are 95% confidence intervals. Figure S8 Forest plot of sensitivity and specificity of 17 individual studies for s-p53-antibody in the diagnosis of hepatocellular carcinoma. The point estimates of sensitivity/specificity from each study ar [file pone.0099255.s001.zip › supplementary materials--Dec/Fig S8.bmp]

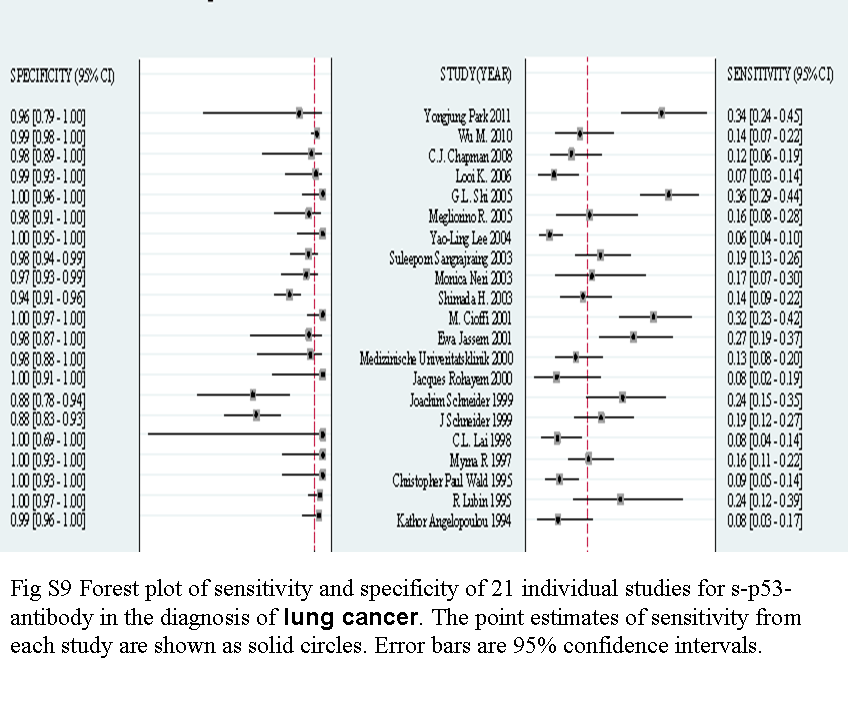

Supplement: File S1 — Supporting figures, tables and references. Figure S1 Methodological quality graph: review authors' judgments about each methodological quality item presented as percentages across all included studies. (PDF). Figure S2 Forest plot of sensitivity and specificity of 5 individual studies for s-p53-antibody in the diagnosis of bladder cancer. The point estimates of sensitivity/specificity from each study are shown as solid circles. Error bars are 95% confidence intervals. Figure S3 Forest plot of sensitivity and specificity of 12 individual studies for s-p53-antibody in the diagnosis of breast cancer. The point estimates of sensitivity/specificity from each study are shown as solid circles. Error bars are 95% confidence intervals. Figure S4 Forest plot of sensitivity and specificity of 16 individual studies for s-p53-antibody in the diagnosis of colorectal cancer. The point estimates of sensitivity/specificity from each study are shown as solid circles. Error bars are 95% confidence intervals. Figure S5 Forest plot of sensitivity and specificity of 15 individual studies for s-p53-antibody in the diagnosis of EC. The point estimates of sensitivity/specificity from each study are shown as solid circles. Error bars are 95% confidence intervals. Figure S6 Forest plot of sensitivity and specificity of 6 individual studies for s-p53-antibody in the diagnosis of gastric cancer. The point estimates of sensitivity/specificity from each study are shown as solid circles. Error bars are 95% confidence intervals. Figure S7 Forest plot of sensitivity and specificity of 7 individual studies for s-p53-antibody in the diagnosis of head and neck cancer. The point estimates of sensitivity/specificity from each study are shown as solid circles. Error bars are 95% confidence intervals. Figure S8 Forest plot of sensitivity and specificity of 17 individual studies for s-p53-antibody in the diagnosis of hepatocellular carcinoma. The point estimates of sensitivity/specificity from each study ar [file pone.0099255.s001.zip › supplementary materials--Dec/Fig S9.bmp]

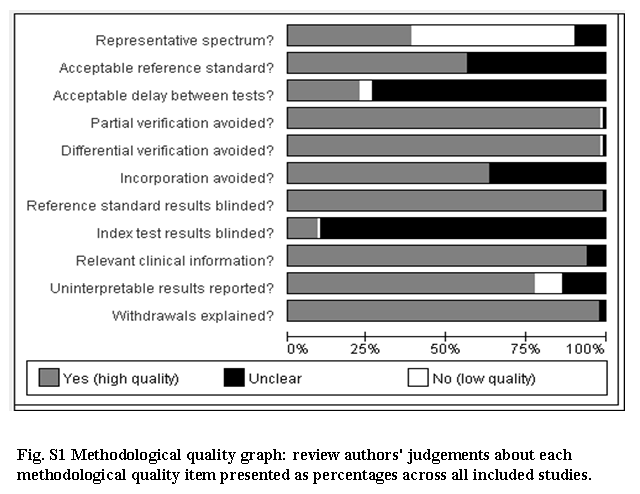

Supplement: File S1 — Supporting figures, tables and references. Figure S1 Methodological quality graph: review authors' judgments about each methodological quality item presented as percentages across all included studies. (PDF). Figure S2 Forest plot of sensitivity and specificity of 5 individual studies for s-p53-antibody in the diagnosis of bladder cancer. The point estimates of sensitivity/specificity from each study are shown as solid circles. Error bars are 95% confidence intervals. Figure S3 Forest plot of sensitivity and specificity of 12 individual studies for s-p53-antibody in the diagnosis of breast cancer. The point estimates of sensitivity/specificity from each study are shown as solid circles. Error bars are 95% confidence intervals. Figure S4 Forest plot of sensitivity and specificity of 16 individual studies for s-p53-antibody in the diagnosis of colorectal cancer. The point estimates of sensitivity/specificity from each study are shown as solid circles. Error bars are 95% confidence intervals. Figure S5 Forest plot of sensitivity and specificity of 15 individual studies for s-p53-antibody in the diagnosis of EC. The point estimates of sensitivity/specificity from each study are shown as solid circles. Error bars are 95% confidence intervals. Figure S6 Forest plot of sensitivity and specificity of 6 individual studies for s-p53-antibody in the diagnosis of gastric cancer. The point estimates of sensitivity/specificity from each study are shown as solid circles. Error bars are 95% confidence intervals. Figure S7 Forest plot of sensitivity and specificity of 7 individual studies for s-p53-antibody in the diagnosis of head and neck cancer. The point estimates of sensitivity/specificity from each study are shown as solid circles. Error bars are 95% confidence intervals. Figure S8 Forest plot of sensitivity and specificity of 17 individual studies for s-p53-antibody in the diagnosis of hepatocellular carcinoma. The point estimates of sensitivity/specificity from each study ar [file pone.0099255.s001.zip › supplementary materials--Dec/Figure S1.TIF]
