# Supplementary material for: Assessment of the Potential Diagnostic Value of Serum p53 Antibody for Cancer: A Meta-Analysis
Source: PLoS One. 2014 Jun 9;9(6):e99255. doi: 10.1371/journal.pone.0099255 (PMC4049633; doi:10.1371/journal.pone.0099255)
Supplement: Checklist S1 — PRISMA Checklist. (DOC) [file pone.0099255.s002.doc]

| **Section/topic** | **#** | **Checklist item** | **Reported on page #** |
| --- | --- | --- | --- |
| **TITLE** | | |  |
| Title | 1 | Assessment of the potenial diagnostic value of serum p53 antibody for cancer: a meta-analysis | #1 |
| **ABSTRACT** | | |  |
| Structured summary | 2 | Background: Mutant p53 protein overexpression has been reported to induce serum antibodies against p53. We assessed the diagnostic precision of serum p53 (s-p53) antibodies for diagnosis of cancer patients and compared s-p53 antibody levels in different types of cancers.Methods: We systematically searched PubMed and Embase, through May 31, 2012, without language restriction. Studies were assessed for quality using QUADAS (quality assessment of studies of diagnostic accuracy). The positive likelihood ratio (PLR) and negative likelihood ratio (NLR) were pooled separately and compared with overall accuracy measures using diagnostic odds ratios (DORs) and symmetric summary receiver operating characteristic (SROC) curves. Meta regression and subgroup analyses were done, and heterogeneity and publication bias were assessed. Results: Of 1089 studies initially identified, 100 eligible studies with 23 different types of tumor met the inclusion criteria for the meta-analysis (cases=15953, controls=8694). However, we could conduct independent meta analysis on only 13 of 36 types of tumors. Approximately 56% (56/100) of the included studies were of high quality (QUADAS score≥8). The summary estimates for quantitative analysis of serum p53 antibody in the diagnosis of cancers were: PLR 12.01 (95% CI: 8.31-17.35), NLR 0.80 (95%CI: 0.77-0.83) and DOR 14.99 (95% CI: 10.27-21.88). However, for the 13 types of cancers on which meta-analysis was conducted, the ranges for PLR (3.62-21.56), NLR (0.74-0.97), DOR (3.74-27.61), AUC (0.31-0.81), and positive rate (4.47%-28.36%) indicated significant heterogeneity. We found that breast, colorectal, esophageal, gastric, hepatic, lymphoma, lung and ovarian cancer had relatively reasonable diagnostic accuracy. The remaining results of the five types of cancers suggested that s-p53 antibody had limited value.  Conclusions: The current evidence suggests that s-p53 antibody has potential diagnostic value for cancer, especially for breast, colorectal, esophageal, gastric, hepatic, lymphoma, lung and ovarian cancer. The results showed that s-p53 antibody had high correlation with cancers. | #2 |
| INTRODUCTION | | |  |
| Rationale | 3 | Cancer has long been recognized as a multi-step process that involves not only genetic changes conferring growth advantage, but also factors that disrupt regulation of growth and differentiation. It is possible that some of these factors could be identiﬁed with the aid of auto-antibodies arising during tumorigenesis. Mutations in the tumor suppressor gene p53 are the most commonly observed genetic abnormalities in human cancers . The protein product of the p53 gene is a nuclear phosphoprotein expressed in normal cells. In the serum of healthy subjects, the presence of p53 protein and anti-p53 antibodies are extremely rare . Mutations in this gene cause an accumulation of non-functional proteins, due to increased stability and a longer half-life of several hours compared with the 20 min half-life for wild-type p53. The accumulated protein then acts as an antigen, with subsequent development of antibodies (anti-p53 antibodies), which are detectable in tissues, sloughed cells, blood, and other body ﬂuids . With the development of molecular biotechnology, a highly specific autoantibody response in systemic autoimmune diseases generally predicts the biologic phenotype of the disease, making autoantibodies clinically valuable and diagnostically useful . Hence, there is a great need for identification of novel non-invasive diagnostic methods for tumor detection. | #3, #4  Introduction |
| Objectives | 4 | Our objective was to obtain the best estimates of the diagnostic accuracy of serum p53 (s-p53) antibody for detection of cancers, and to make comparisons about the diagnostic value of s-p53 antibody in different types of cancers by performing a systematic review and meta-analysis. | #4, #5  Introduction |
| METHODS | | |  |
| Protocol and registration | 5 | No protocol and registration . |  |
| Eligibility criteria | 6 | Inclusion criteria for the primary studies were as follows: (i) participants: all cases must have been diagnosed by pathologic examination of biopsied specimens, serum must have been collected for anti-p53 analysis before any treatment, e.g. chemotherapy or radiotherapy, and controls were without other cancers, (ii) index test: studies evaluated the diagnostic value of s-p53 antibody in cancer patients, (iii) outcome: studies reported the positive values of the cases and controls, and the results of an individual study on diagnostic accuracy could be summarized in a 2×2 table, (iv) study design: No restrictions were made with respect to study design (cross sectional, case control, cohort study) or data collection (prospective or retrospective). | #5, #6 Search strategy and study selection |
| Information sources | 7 | We searched PubMed and EMBASE to identify suitable studies prior to 31st May, 2012. No start data limit was applied. | #5Search strategy and study selection |
| Search | 8 | #1 Neoplasms [Mesh]  #2 Tumor OR tumour OR neoplasm OR neoplasms OR cancer OR melanoma OR carcinoma [all fields]  #3 #1 or #2  #4 P53 or TP53 [all fields]  #5 Antibody or autoantibody [all fields]  #6 #4 and #5  #7 Blood OR serum OR serological OR seropositive OR seropositivity OR serum antibody OR sera OR plasma[all fields]  #8 #3 and #6 and #7 limits: humans | #5 Table S2 Search strategy in PubMed. |
| Study selection | 9 | Two reviewers (J Zhang and ZW Xu) independently inspected the title and abstract of each citation to identify those studies that were likely to report the diagnostic value of serum p53 (s-p53) antibody, and then obtained the full text. Disagreements about study selection were resolved by consensus. The full text was retrieved for articles that could not be excluded based on title and abstract to determine inclusion. | #5 Search strategy and study selection |
| Data collection process | 10 | The following characteristics studies were extracted: (i) basic information: conductor, study ID and study details (first author, year of publication, country of study, tumor type), (ii) study eligibility: based on inclusion/exclusion criteria to assess again and to record the reason for the excluded studies, (iii) methods of the study characteristics: participants’ inclusion/exclusion criteria, ethnicity, disease stage, histology stage, standard reference, type of control, (iv) index tests: the extraction time and storage temperature of the sample, assay method, cut-off value, blind (single-blind or doubled-blind), a detailed report of the assay procedure, (v) outcome: the positive value of the cases and controls, and other comparison data (e.g. mean age, sex ratio, smoking, drinking) between cases and controls. | #7 Data extraction and management |
| Data items | 11 | outcome: the positive value of the cases and controls, and other comparison data (e.g. mean age, sex ratio, smoking, drinking) between cases and controls. | #7 Data extraction and management |
| Risk of bias in individual studies | 12 | In all 100 studies, cancer patients diagnosed by histology were regarded as positive. However, the negative controls without cancer who were healthy or had benign disease were not diagnosed by histology. In addition, most of the studies did not report whether the investigators were blinded. Therefore, such non-strict designs could exaggerate the diagnostic accuracy and lead to bias due to unfavorable representation of the participants. | #14 Discussion |
| Summary measures | 13 | The accuracy measure used was the diagnostic odds ratio (DOR) computed by the Moses’s constant of linear method. Summary receiver operating characteristic curves were used to summarize overall test performance, and the area under the SROC curve (AUC) was calculated. We used a chi-squared test to detect statistically significant heterogeneity. Between-study heterogeneity was assessed using I². To detect cut-off threshold effects, the relationship between sensitivity and specificity was evaluated by using the Spearman correlation coefficient r. | #8, #9 Statistical analyses |
| Synthesis of results | 14 | For all of the cancers included in the 100 studies, the pooled DOR was 14.99 (95%CI: 10.27- 21.88), indicating that s-p53 antibody could be a useful biomarker for cancer patient diagnosis. There appeared to be qualitative evidence for heterogeneity between studies (Cochran Q=1.5e+10, I²=100%). Fig. 2 presents the symmetrical SROC of s-p53 antibody, showing an AUC of 0.71, indicating that s-p53 antibodies had reasonable accuracy in terms of differential diagnosis in cases of cancer. Of the 100 eligible studies, sensitivity and specificity ranged from 2.90%-68.30% and 67.30%-100%, respectively. In the present study, a pooled PLR of 12.01 (95% CI: 8.31-17.35) suggests that patients with cancer have 12-fold higher chance of being s-p53 antibody-positive compared with patients without cancer. Also, there was heterogeneity between PLRs, with Cochran Q=261.19 and I²=54.37%. Similarly, we found significant heterogeneity for all of the eligible studies regarding NLR, with Cochran Q =1175.24 and I²=91.66%. The pooled negative likelihood ratio was 0.80 (95% CI: 0.77-0.83), indicating that patients without cancer have a 1.25-fold higher chance of being s-p53 antibody-negative compared with patients with cancer. | #13,#14  Diagnostic accuracy |

Page 1 of 2

| **Section/topic** | **#** | **Checklist item** | **Reported on page #** |
| --- | --- | --- | --- |
| Risk of bias across studies | 15 | Quality assessment based on QUADAS guidelines was conducted on all 100 studies included for systematic review. (Fgure S1) . | #12 Methodological quality of included studies |
| Additional analyses | 16 | The meta-regression and sub-group analyses were used to explore the overall heterogeneity and the possible sources of heterogeneity. | #15 Possible sources of heterogeneity |
| RESULTS | | |  |
| Study selection | 17 | Fig.1 Flow chart of study selection by using electronic database and other sources. | #11 |
| Study characteristics | 18 | Table S1. Main characteristics of the 100 eligible studies | #11 |
| Risk of bias within studies | 19 | Sensitivity analysis was conducted in terms of statistical analysis methods, sample size, and study design,respectively. However, the results produced no obvious changes. Publication bias is assessed visually by using a scatter plot of the inverse of the square root of the effective sample size (1/ESS1/2) versus the diagnostic log odds ratio (lnDOR) ,the results showed no publication bias in this meta-analysis (p=0.000) . | #16 Sensitivity analysis and publication bias |
| Results of individual studies | 20 | The data of 100 individual studies were not shown. |  |
| Synthesis of results | 21 | Table 1 Pooled diagnostic accuracy of s-p53 antibody for detection of 13 types of cancer.  Table 2 Possible sources of heterogeneity of sub-group analysis. | #13, #14 |
| Risk of bias across studies | 22 | Figure 2 Funnel plot for the assessment of potential bias in s-p53 antibody assays. The funnel graph plots the DOR (diagnostic odds ratio) against the 1/root (effective sample size). The dotted line is the regression line. The result of the test for publication bias showed publication bias (p=0.000). (PPT) | #16, #17 |
| Additional analysis | 23 | The meta-regression and sub-group analyses were used to explore the overall heterogeneity and the possible sources of heterogeneity(Table 2), and the sensitive analysis was used to detect the validity of the results. | #26  Possible sources of heterogeneity |
| DISCUSSION | | |  |
| Summary of evidence | 24 | In a systematic review of the published literature, we find that patients with cancer have a higher chance of being s-p53 antibody-positive compared with patients without cancer, and that the ratio of the odds of a positive test result among cancer patients is approximately 15-fold the odds of a positive test result among non-cancer individuals. Furthermore, the ratio of the odds of a positive test result among cancer patients is approximately 7-fold the odds of a positive test result for benign disease. In brief, the positive frequency of s-p53 antibody in most of the cancer patients is higher than in healthy and benign controls. Therefore, a positive s-p53 antibody test is diagnostic of cancer. | #17  Disscussion |
| Limitations | 25 | The present study has several limitations: First, we did not calculate the diagnostic accuracy for the early stage (stage I-II) cancers because sufficient raw data was not provided. Although we aimed to evaluate the diagnostic value of s-p53 antibodies for the early diagnosis of the cancer, cancer patients regardless of disease stage were used to evaluate the diagnostic power because of the limitation of information. Primary data were unavailable for investigation of elevated or decreased s-p53 antibody values as a function of tumor type, histology, age, or degree. Second, 54 of the 100 included studies used healthy controls, whereas only 20 studies used benign disease, and 20 studies used both healthy controls and benign controls, which strongly exaggerated the diagnostic accuracy. A higher value of DOR is obtained between cancer patients and health controls (DOR, 20.56, 95%CI: 11.79-35.83) than between cancer patients and benign disease (DOR, 6.55, 95%CI: 4.01-10.72). Although the non-restricted design could overestimate the discrimination power of s-p53-antibodies in cancer, the meta-analysis based on comprehensive, large sample quantitative assessments provides more convincing evidence. Indeed, evidence for the diagnostic value of s-p53 antibody for cancer is compelling in that the PLR, DOR values were all larger than five. Thirdly, systematic reviewers are advised to use comprehensive searches to attempt to locate all relevant studies . | #20  Disscussion |
| Conclusions | 26 | In conclusion, the current evidence suggests that s-p53 antibody is a useful biomarker for cancer diagnosis, especially for breast, colorectal, esophageal, gastric, hepatic, lymphoma, lung and ovarian cancers. Significantly, there few individuals in healthy controls display s-p53 antibodies. However, it is not uncommon that the frequency of s-p53 antibody-positive individuals is different between most types of cancer patients and healthy controls. Patients with cancer have a higher chance of being s-p53 antibody-positive compared with patients without cancer. | #21  Disscussion |
| **FUNDING** | | |  |
| Funding | 27 | No any funding and support . |  |

*From:*  Moher D, Liberati A, Tetzlaff J, Altman DG, The PRISMA Group (2009). Preferred Reporting Items for Systematic Reviews and Meta-Analyses: The PRISMA Statement. PLoS Med 6(6): e1000097. doi:10.1371/journal.pmed1000097

For more information, visit: **www.prisma-statement.org**.

Page 2 of 2
