# Supplementary material for: Assessment of the Potential Diagnostic Value of Serum p53 Antibody for Cancer: A Meta-Analysis
Source: PLoS One. 2014 Jun 9;9(6):e99255. doi: 10.1371/journal.pone.0099255 (PMC4049633; doi:10.1371/journal.pone.0099255)
Supplement: File S1 — Supporting figures, tables and references. Figure S1 Methodological quality graph: review authors' judgments about each methodological quality item presented as percentages across all included studies. (PDF). Figure S2 Forest plot of sensitivity and specificity of 5 individual studies for s-p53-antibody in the diagnosis of bladder cancer. The point estimates of sensitivity/specificity from each study are shown as solid circles. Error bars are 95% confidence intervals. Figure S3 Forest plot of sensitivity and specificity of 12 individual studies for s-p53-antibody in the diagnosis of breast cancer. The point estimates of sensitivity/specificity from each study are shown as solid circles. Error bars are 95% confidence intervals. Figure S4 Forest plot of sensitivity and specificity of 16 individual studies for s-p53-antibody in the diagnosis of colorectal cancer. The point estimates of sensitivity/specificity from each study are shown as solid circles. Error bars are 95% confidence intervals. Figure S5 Forest plot of sensitivity and specificity of 15 individual studies for s-p53-antibody in the diagnosis of EC. The point estimates of sensitivity/specificity from each study are shown as solid circles. Error bars are 95% confidence intervals. Figure S6 Forest plot of sensitivity and specificity of 6 individual studies for s-p53-antibody in the diagnosis of gastric cancer. The point estimates of sensitivity/specificity from each study are shown as solid circles. Error bars are 95% confidence intervals. Figure S7 Forest plot of sensitivity and specificity of 7 individual studies for s-p53-antibody in the diagnosis of head and neck cancer. The point estimates of sensitivity/specificity from each study are shown as solid circles. Error bars are 95% confidence intervals. Figure S8 Forest plot of sensitivity and specificity of 17 individual studies for s-p53-antibody in the diagnosis of hepatocellular carcinoma. The point estimates of sensitivity/specificity from each study ar [file pone.0099255.s001.zip › supplementary materials--Dec/Included studies.doc]

***Included studies (reference):***

## *1. Wu M, Mao C, Chen Q, Cu XW, Zhang WS (2010) Serum p53 protein and anti-p53 antibodies are associated with increased cancer risk: a case-control study of 569 patients and 879 healthy controls. Mol Biol Rep 37: 339-343.*

## *2. Caron de Fromentel C, May-Levin F, Mouriesse H, Lemerle J, Chandrasekaran K, et al. (1987) Presence of circulating antibodies against cellular protein p53 in a notable proportion of children with B-cell lymphoma. Int J Cancer 39: 185-189.*

## *3. Labrecque S, Naor N, Thomson D, Matlashewski G (1993) Analysis of the anti-p53 antibody response in cancer patients. Cancer Res 53: 3468-3471.*

## *4. Volkmann M, Muller M, Hofmann WJ, Meyer M, Hagelstein J, et al. (1993) The humoral immune response to p53 in patients with hepatocellular carcinoma is specific for malignancy and independent of the alpha-fetoprotein status. Hepatology 18: 559-565.*

## *5. Angelopoulou K, Diamandis EP, Sutherland DJ, Kellen JA, Bunting PS (1994) Prevalence of serum antibodies against the p53 tumor suppressor gene protein in various cancers. Int J Cancer 58: 480-487.*

## *6. Green JA, Mudenda B, Jenkins J, Leinster SJ, Tarunina M, et al. (1994) Serum p53 auto-antibodies: incidence in familial breast cancer. Eur J Cancer 30A: 580-584.*

## *7. Lahdeaho ML, Lehtinen T, Aine R, Hakala T, Lehtinen M (1994) Antibody response to adenovirus E1b-derived synthetic peptides and serum levels of p53 in patients with gastrointestinal and other malignant lymphomas. J Med Virol 43: 393-396.*

## *8. Marxsen J, Schmiegel W, Roder C, Harder R, Juhl H, et al. (1994) Detection of the anti-p53 antibody response in malignant and benign pancreatic disease. Br J Cancer 70: 1031-1034.*

## *9. Mudenda B, Green JA, Green B, Jenkins JR, Robertson L, et al. (1994) The relationship between serum p53 autoantibodies and characteristics of human breast cancer. Br J Cancer 69: 1115-1119.*

## *10. Green JA, Robertson LJ, Campbell IR, Jenkins J (1995) Expression of the p53 gene and presence of serum autoantibodies in ovarian cancer: correlation with differentiation. Cancer Detect Prev 19: 151-155.*

## *11. Laurent-Puig P, Lubin R, Semhoun-Ducloux S, Pelletier G, Fourre C, et al. (1995) Antibodies against p53 protein in serum of patients with benign or malignant pancreatic and biliary diseases. Gut 36: 455-458.*

## *12. Lubin R, Schlichtholz B, Teillaud JL, Garay E, Bussel A, et al. (1995) p53 antibodies in patients with various types of cancer: assay, identification, and characterization. Clin Cancer Res 1: 1463-1469.*

## *13. Wild CP, Ridanpaa M, Anttila S, Lubin R, Soussi T, et al. (1995) p53 antibodies in the sera of lung cancer patients: comparison with p53 mutation in the tumour tissue. Int J Cancer 64: 176-181.*

## *14. Coomber D, Hawkins NJ, Clark M, Meagher A, Ward RL (1996) Characterisation and clinicopathological correlates of serum anti-p53 antibodies in breast and colon cancer. J Cancer Res Clin Oncol 122: 757-762.*

## *15. Gessner CE, Baillie J (1996) P53 levels in the evaluation of malignant/benign pancreaticobiliary disease: hope or hype? Am J Gastroenterol 91: 401-402.*

## *16. Maass JD, Gottschlich S, Lippert BM, Niemann AM, Gorogh T, et al. (1996) [Antibody formation to cellular p53 protein in patients with squamous cell carcinomas of the upper respiratory and digestive system]. Laryngorhinootologie 75: 53-56.*

## *17. Raedle J, Oremek G, Welker M, Roth WK, Caspary WF, et al. (1996) p53 autoantibodies in patients with pancreatitis and pancreatic carcinoma. Pancreas 13: 241-246.*

## *18. Ryder SD, Rizzi PM, Volkmann M, Metivier E, Pereira LM, et al. (1996) Use of specific ELISA for the detection of antibodies directed against p53 protein in patients with hepatocellular carcinoma. J Clin Pathol 49: 295-299.*

## *19. Shibata Y, Kotanagi H, Andoh H, Koyama K, Itoh H, et al. (1996) Detection of circulating anti-p53 antibodies in patients with colorectal carcinoma and the antibody's relation to clinical factors. Dis Colon Rectum 39: 1269-1274.*

## *20. Angelopoulou K, Stratis M, Diamandis EP (1997) Humoral immune response against p53 protein in patients with colorectal carcinoma. Int J Cancer 70: 46-51.*

## *21. Friedrich RE, Bartel-Friedrich S, Plambeck K, Bahlo M, Klapdor R (1997) P53 auto-antibodies in the sera of patients with oral squamous cell carcinoma. Anticancer Res 17: 3183-3184.*

## *22. Hammel P, Boissier B, Chaumette MT, Piedbois P, Rotman N, et al. (1997) Detection and monitoring of serum p53 antibodies in patients with colorectal cancer. Gut 40: 356-361.*

## *23. Kaur J, Srivastava A, Ralhan R (1997) Serum p53 antibodies in patients with oral lesions: correlation with p53/HSP70 complexes. Int J Cancer 74: 609-613.*

## *24. Maass J, Gottschlich S, Goeroegh T, Lippert J, Werner J (1997) Head and neck cancer and p53-immunogenicity. Anticancer Res 17: 2873-2874.*

## *25. Rosenfeld MR, Malats N, Schramm L, Graus F, Cardenal F, et al. (1997) Serum anti-p53 antibodies and prognosis of patients with small-cell lung cancer. J Natl Cancer Inst 89: 381-385.*

## *26. Wollenberg B, Jan N, Pitzke P, Reiter W, Stieber P (1997) Anti-p53 antibodies in serum of smokers and head and neck cancer patients. Anticancer Res 17: 413-418.*

## *27. Wurl P, Weigmann F, Meye A, Fittkau M, Rose U, et al. (1997) Detection of p53 autoantibodies in sera of gastric cancer patients and their prognostic relevance. Scand J Gastroenterol 32: 1147-1151.*

## *28. Cawley HM, Meltzer SJ, De Benedetti VM, Hollstein MC, Muehlbauer KR, et al. (1998) Anti-p53 antibodies in patients with Barrett's esophagus or esophageal carcinoma can predate cancer diagnosis. Gastroenterology 115: 19-27.*

## *29. Edis C, Kahler C, Klotz W, Herold M, Feichtinger H, et al. (1998) A comparison between alpha-fetoprotein and p53 antibodies in the diagnosis of hepatocellular carcinoma. Transplant Proc 30: 780-781.*

## *30. Hallak R, Mueller J, Lotter O, Gansauge S, Gansauge F, et al. (1998) p53 genetic alterations, protein expression and autoantibodies in human colorectal carcinoma: A comparative study. Int J Oncol 12: 785-791.*

## *31. Lai CL, Tsai CM, Tsai TT, Kuo BI, Chang KT, et al. (1998) Presence of serum anti-p53 antibodies is associated with pleural effusion and poor prognosis in lung cancer patients. Clin Cancer Res 4: 3025-3030.*

## *32. Lang C, Unteregger G, Kartarius S, Gunther J, Bonkhoff H, et al. (1998) p53 autoantibodies in patients with urological tumours. Br J Urol 82: 721-726.*

## *33. Ralhan R, Nath N, Agarwal S, Mathur M, Wasylyk B, et al. (1998) Circulating p53 antibodies as early markers of oral cancer: correlation with p53 alterations. Clin Cancer Res 4: 2147-2152.*

## *34. Shiota G, Ishida M, Noguchi N, Takano Y, Oyama K, et al. (1998) Clinical significance of serum P53 antibody in patients with gastric cancer. Res Commun Mol Pathol Pharmacol 99: 41-51.*

## *35. Sobti RC, Parashar K (1998) A study on p53 protein and anti-p53 antibodies in the sera of patients with oesophageal cancer. Mutat Res 422: 271-277.*

## *36. Lenner P, Wiklund F, Emdin SO, Arnerlov C, Eklund C, et al. (1999) Serum antibodies against p53 in relation to cancer risk and prognosis in breast cancer: a population-based epidemiological study. Br J Cancer 79: 927-932.*

## *37. Maehara Y, Kakeji Y, Watanabe A, Baba H, Kusumoto H, et al. (1999) Clinical implications of serum anti-p53 antibodies for patients with gastric carcinoma. Cancer 85: 302-308.*

## *38. Mayerhofer K, Hefler L, Schindl M, Concin N, Frischmuth K, et al. (1999) P53 antibody response in patients with vulvar cancer. Anticancer Res 19: 2323-2326.*

## *39. Mayerhofer K, Tempfer C, Kucera E, Hefler L, Zeisler H, et al. (1999) Humoral p53 antibody response is a prognostic parameter in ovarian cancer. Anticancer Res 19: 875-878.*

## *40. Saffroy R, Lelong JC, Azoulay D, Salvucci M, Reynes M, et al. (1999) Clinical significance of circulating anti-p53 antibodies in European patients with hepatocellular carcinoma. Br J Cancer 79: 604-610.*

## *41. Sanchez-Carbayo M, Chulia MT, Niveiro M, Aranda I, Mira A, et al. (1999) Autoantibodies against P53 protein in patients with transitional cell carcinoma of the bladder. Anticancer Res 19: 3531-3537.*

## *42. Schneider J, Presek P, Braun A, Bauer P, Konietzko N, et al. (1999) p53 protein, EGF receptor, and anti-p53 antibodies in serum from patients with occupationally derived lung cancer. Br J Cancer 80: 1987-1994.*

## *43. Schneider J, Presek P, Braun A, Woitowitz HJ (1999) Serum levels of pantropic p53 protein and EGF-receptor, and detection of anti-p53 antibodies in former uranium miners (SDAG Wismut). Am J Ind Med 36: 602-609.*

## *44. Wu CW, Lin YY, Chen GD, Chi CW, Carbone DP, et al. (1999) Serum anti-p53 antibodies in gastric adenocarcinoma patients are associated with poor prognosis, lymph node metastasis and poorly differentiated nuclear grade. Br J Cancer 80: 483-488.*

## *45. Fellay-Reynier I, Orsiere T, Sari-Minodier I, Auquier P, Zattara-Cannoni H, et al. (2000) Evaluation of micronucleated lymphocytes, constitutional karyotypes and anti-p53 antibodies in 21 children with various malignancies. Mutat Res 467: 31-39.*

## *46. Hagiwara N, Onda M, Miyashita M, Sasajima K (2000) Detection of circulating anti-p53 antibodies in esophageal cancer patients. J Nippon Med Sch 67: 110-117.*

## *47. Mack U, Ukena D, Montenarh M, Sybrecht GW (2000) Serum anti-p53 antibodies in patients with lung cancer. Oncol Rep 7: 669-674.*

## *48. Metcalfe S, Wheeler TK, Picken S, Negus S, Jo Milner A (2000) P53 autoantibodies in 1006 patients followed up for breast cancer. Breast Cancer Res 2: 438-443.*

## *49. Ralhan R, Arora S, Chattopadhyay TK, Shukla NK, Mathur M (2000) Circulating p53 antibodies, p53 gene mutational profile and product accumulation in esophageal squamous-cell carcinoma in India. Int J Cancer 85: 791-795.*

## *50. Rohayem J, Diestelkoetter P, Weigle B, Oehmichen A, Schmitz M, et al. (2000) Antibody response to the tumor-associated inhibitor of apoptosis protein survivin in cancer patients. Cancer Res 60: 1815-1817.*

## *51. Shimada H, Nakajima K, Sakamoto K, Takeda A, Hori S, et al. (2000) Existence of serum p53 antibodies in cyclosporine A-treated transplant patients: possible detection of p53 protein over-expression. Transplant Proc 32: 1779.*

## *52. Shimada H, Takeda A, Arima M, Okazumi S, Matsubara H, et al. (2000) Serum p53 antibody is a useful tumor marker in superficial esophageal squamous cell carcinoma. Cancer 89: 1677-1683.*

## *53. Wunderlich H, Hindermann W, Huller M, Reichelt O, Werner W, et al. (2000) The correlation of p53 protein overexpression and p53 antibodies in serum of patients with transitional cell carcinoma of the urinary bladder. Urol Int 64: 13-17.*

## *54. Broll R, Duchrow M, Oevermann E, Wellm C, Schwandner O, et al. (2001) p53 autoantibodies in sera of patients with a colorectal cancer and their association to p53 protein concentration and p53 immunohistochemistry in tumor tissue. Int J Colorectal Dis 16: 22-27.*

## *55. Charuruks N, Tangkijvanich P, Voravud N, Chatsantikul R, Theamboonlers A, et al. (2001) Clinical significance of p53 antigen and anti-p53 antibodies in the sera of hepatocellular carcinoma patients. J Gastroenterol 36: 830-836.*

## *56. Chow V, Yuen APW, Lam KY, Ho WK, Wei WI (2001) Prognostic significance of serum p53 protein and p53 antibody in patients with surgical treatment for head and neck squamous cell carcinoma. Head Neck 23: 286-291.*

## *57. Cioffi M, Vietri MT, Gazzerro P, Magnetta R, D'Auria A, et al. (2001) Serum anti-p53 antibodies in lung cancer: comparison with established tumor markers. Lung Cancer 33: 163-169.*

## *58. Jassem E, Bigda J, Dziadziuszko R, Schlichtholz B, Le Roux D, et al. (2001) Serum p53 antibodies in small cell lung cancer: the lack of prognostic relevance. Lung Cancer 31:* *59. Jezersek B, Rudolf Z, Novakovic S (2001) The circulating auto-antibodies to p53 protein in the follow-up of lymphoma patients. Oncol Rep 8: 77-81.*

## *60. Kozlowski M, Kovalchuk O, Niklinski J, Chyczewski L, Staroslawska E, et al. (2001) Circulating anti-p53 antibodies in esophageal cancer patients. Folia Histochem Cytobiol 39 Suppl 2: 173-174.*

## *61. Moch C, Moysan A, Lubin R, de la Salmoniere P, Soufir N, et al. (2001) Divergence between the high rate of p53 mutations in skin carcinomas and the low prevalence of anti-p53 antibodies. Br J Cancer 85: 1883-1886.*

## *62. Numa F, Umayahara K, Suehiro Y, Hirakawa H, Nawata S, et al. (2001) Serum anti-p53 antibodies in uterine and ovarian cancer: association with dna sequence copy number abnormalities. Tumour Biol 22: 162-168.*

## *63. Wurl P, Fittkau M, Meye A, Bartel F, Schmidt H, et al. (2001) Low detection rate of p53 antibodies in sera of soft tissue sarcoma patients. Cancer Lett 170: 199-205.*

## *64. Hofele C, Schwager-Schmitt M, Volkmann M (2002) [Prognostic value of antibodies against p53 in patients with oral squamous cell carcinoma--five years survival rate]. Laryngorhinootologie 81: 342-345.*

## *65. Hogdall EV, Hogdall CK, Blaakaer J, Heegaard NH, Glud E, et al. (2002) P53 autoantibodies in sera from Danish ovarian cancer patients and their correlation with clinical data and prognosis. APMIS 110: 545-553.*

## *66. Ohshio G, Suwa H, Imamura M (2002) Clinical implication of anti-p53 antibodies and p53-protein in pancreatic disease. Int J Gastrointest Cancer 31: 129-135.*

## *67. Sakai H (2002) [Detection of serum anti-p53 antibodies in patients with various types of cancer]. Rinsho Byori 50: 90-92.*

## *68. Shimada H, Nabeya Y, Okazumi S, Matsubara H, Funami Y, et al. (2002) Prognostic significance of serum p53 antibody in patients with esophageal squamous cell carcinoma. Surgery 132: 41-47.*

## *69. Fonseca RF, Kawamura MT, Oliveira JA, Teixeira A, Alves G, et al. (2003) Anti-P53 antibodies in Brazilian brain tumor patients. Genet Mol Res 2: 185-190.*

## *70. Neri M, Betta P, Marroni P, Filiberti R, Cafferata M, et al. (2003) Serum anti-p53 autoantibodies in pleural malignant mesothelioma, lung cancer and non-neoplastic lung diseases. Lung Cancer 39: 165-172.*

## *71. Sangrajrang S, Sornprom A, Chernrungroj G, Soussi T (2003) Serum p53 antibodies in patients with lung cancer: correlation with clinicopathologic features and smoking. Lung Cancer 39: 297-301.*

## *72. Shimada H, Ochiai T, Nomura F (2003) Titration of serum p53 antibodies in 1,085 patients with various types of malignant tumors: a multiinstitutional analysis by the Japan p53 Antibody Research Group. Cancer 97: 682-689.*

## *73. Lechpammer M, Lukac J, Lechpammer S, Kovacevic D, Loda M, et al. (2004) Humoral immune response to p53 correlates with clinical course in colorectal cancer patients during adjuvant chemotherapy. Int J Colorectal Dis 19: 114-120.*

## *74. Lee YL, Shih CM, Chiou HL, Shiau MY, Chang GC, et al. (2004) Alteration of autoantibodies against p53 in Taiwanese lung cancer patients undergoing chemotherapy. Clin Chim Acta 349: 87-96.*

## *75. Shaarawy M, Sheiba M (2004) Diagnostic and prognostic significance of circulating tumor suppressor gene p53 autoantibodies in patients with gestational trophoblastic tumors. Acta Oncol 43: 43-48.*

## *76. Wang MH, Li YP, Wang XD, Zhang H, Xiong LH, et al. (2004) [Correlation between serum p53 antibody and chemosensitivity in patient with esophageal cancer]. Ai Zheng 23: 467-470.*

## *77. Himoto T, Kuriyama S, Zhang JY, Chan EK, Kimura Y, et al. (2005) Analyses of autoantibodies against tumor-associated antigens in patients with hepatocellular carcinoma. Int J Oncol 27: 1079-1085.*

## *78. Kato H, Nakajima M, Fukuchi M, Miyazaki T, Manda R, et al. (2005) Serum p53 antibodies in drainage blood from the azygos vein in patients with esophageal carcinoma. Anticancer Res 25: 3231-3235.*

## *79. Megliorino R, Shi FD, Peng XX, Wang X, Chan EK, et al. (2005) Autoimmune response to anti-apoptotic protein survivin and its association with antibodies to p53 and c-myc in cancer detection. Cancer Detect Prev 29: 241-248.*

## *80. Shi GL, Hu XL, Yue SD, Song CX (2005) [The value of serum tumor marker in the diagnosis of lung cancer]. Zhonghua Zhong Liu Za Zhi 27: 299-301.*

## *81. Balogh GA, Mailo DA, Corte MM, Roncoroni P, Nardi H, et al. (2006) Mutant p53 protein in serum could be used as a molecular marker in human breast cancer. Int J Oncol 28: 995-1002.*

## *82. Looi K, Megliorino R, Shi FD, Peng XX, Chen Y, et al. (2006) Humoral immune response to p16, a cyclin-dependent kinase inhibitor in human malignancies. Oncol Rep 16: 1105-1110.*

## *83. Muller M, Meyer M, Schilling T, Ulsperger E, Lehnert T, et al. (2006) Testing for anti-p53 antibodies increases the diagnostic sensitivity of conventional tumor markers. Int J Oncol 29: 973-980.*

## *84. Sainger RN, Shah MH, Desai AA, Shukla SN, Shah PM, et al. (2006) Clinical significance of serum p53 antibodies in oral cancer. Tumori 92: 134-139.*

## *85. Akere A, Otegbayo JA (2007) Evaluation of the pattern and prognostic implications of anti-p53 in hepatocellular carcinoma. Singapore Med J 48: 41-44.*

## *86. Al Kassam D, Álvarez Marcos C, Blanco I, de Los Toyos JR, Luis Llorente J (2007) Valor diagnóstico de los marcadores E-cadherina, MMP-9, MMP-13 activada y de los anticuerpos anti-p53 en el carcinoma escamoso de cabeza y cuello. Med Clin (Barc) 129: 761-765.*

## *87. Mattioni M, Soddu S, Porrello A, D'Alessandro R, Spila A, et al. (2007) Serum anti-p53 antibodies as a useful marker for prognosis of gastric carcinoma. Int J Biol Markers 22: 302-306.*

## *88. Qiu LL, Hua PY, Ye LL, Wang YC, Qiu T, et al. (2007) The detection of serum anti-p53 antibodies from patients with gastric carcinoma in China. Cancer Detect Prev 31: 45-49.*

## *89. Yoshizawa S, Matsuoka K, Inoue N, Takaishi H, Ogata H, et al. (2007) Clinical significance of serum p53 antibodies in patients with ulcerative colitis and its carcinogenesis. Inflamm Bowel Dis 13: 865-873.*

## *90. Zhang JY, Megliorino R, Peng XX, Tan EM, Chen Y, et al. (2007) Antibody detection using tumor-associated antigen mini-array in immunodiagnosing human hepatocellular carcinoma. J Hepatol 46: 107-114.*

## *91. Atta MM, el-Masry SA, Abdel-Hameed M, Baiomy HA, Ramadan NE (2008) Value of serum anti-p53 antibodies as a prognostic factor in Egyptian patients with hepatocellular carcinoma. Clin Biochem 41: 1131-1139.*

## *92. Cai HY, Wang XH, Tian Y, Gao LY, Zhang LJ, et al. (2008) Changes of serum p53 antibodies and clinical significance of radiotherapy for esophageal squamous cell carcinoma. World J Gastroenterol 14: 4082-4086.*

## *93. Chapman CJ, Murray A, McElveen JE, Sahin U, Luxemburger U, et al. (2008) Autoantibodies in lung cancer: possibilities for early detection and subsequent cure. Thorax 63: 228-233.*

## *94. Suppiah A, Alabi A, Madden L, Hartley JE, Monson JR, et al. (2008) Anti-p53 autoantibody in colorectal cancer: prognostic significance in long-term follow-up. Int J Colorectal Dis 23: 595-600.*

## *95. Attallah AM, Shiha GE, Ismail H, Mansy SE, El-Sherbiny R, et al. (2009) Expression of p53 protein in liver and sera of patients with liver fibrosis, liver cirrhosis or hepatocellular carcinoma associated with chronic HCV infection. Clin Biochem 42: 455-461.*

## *96. Gadelhak NA, Gadelhak SA, El-Morsi DA, Abdelaziz MM, Abbas AT, et al. (2009) Prognostic significance of three hepatitis markers (p53 antibodies, vascular endothelial growth factors and alpha fetoprotein) in patients with hepatocellular carcinoma. Hepatogastroenterology 56: 1417-1424.*

## *97. Anderson KS, Wong J, Vitonis A, Crum CP, Sluss PM, et al. (2010) p53 autoantibodies as potential detection and prognostic biomarkers in serous ovarian cancer. Cancer Epidemiol Biomarkers Prev 19: 859-868.*

## *98. Kulic A, Sirotkovic-Skerlev M, Jelisavac-Cosic S, Herceg D, Kovac Z, et al. (2010) Anti-p53 antibodies in serum: relationship to tumor biology and prognosis of breast cancer patients. Med Oncol 27: 887-893.*

## *99. Park Y, Kim Y, Lee JH, Lee EY, Kim HS (2011) Usefulness of serum anti-p53 antibody assay for lung cancer diagnosis. Arch Pathol Lab Med 135: 1570-1575.*

## *100. Tang R, Ko MC, Wang JY, Changchien CR, Chen HH, et al. (2001) Humoral response to p53 in human colorectal tumors: a prospective study of 1,209 patients. International journal of cancer 94: 859-863.*
