# Supplementary material for: Assessment of the Potential Diagnostic Value of Serum p53 Antibody for Cancer: A Meta-Analysis
Source: PLoS One. 2014 Jun 9;9(6):e99255. doi: 10.1371/journal.pone.0099255 (PMC4049633; doi:10.1371/journal.pone.0099255)
Supplement: File S1 — Supporting figures, tables and references. Figure S1 Methodological quality graph: review authors' judgments about each methodological quality item presented as percentages across all included studies. (PDF). Figure S2 Forest plot of sensitivity and specificity of 5 individual studies for s-p53-antibody in the diagnosis of bladder cancer. The point estimates of sensitivity/specificity from each study are shown as solid circles. Error bars are 95% confidence intervals. Figure S3 Forest plot of sensitivity and specificity of 12 individual studies for s-p53-antibody in the diagnosis of breast cancer. The point estimates of sensitivity/specificity from each study are shown as solid circles. Error bars are 95% confidence intervals. Figure S4 Forest plot of sensitivity and specificity of 16 individual studies for s-p53-antibody in the diagnosis of colorectal cancer. The point estimates of sensitivity/specificity from each study are shown as solid circles. Error bars are 95% confidence intervals. Figure S5 Forest plot of sensitivity and specificity of 15 individual studies for s-p53-antibody in the diagnosis of EC. The point estimates of sensitivity/specificity from each study are shown as solid circles. Error bars are 95% confidence intervals. Figure S6 Forest plot of sensitivity and specificity of 6 individual studies for s-p53-antibody in the diagnosis of gastric cancer. The point estimates of sensitivity/specificity from each study are shown as solid circles. Error bars are 95% confidence intervals. Figure S7 Forest plot of sensitivity and specificity of 7 individual studies for s-p53-antibody in the diagnosis of head and neck cancer. The point estimates of sensitivity/specificity from each study are shown as solid circles. Error bars are 95% confidence intervals. Figure S8 Forest plot of sensitivity and specificity of 17 individual studies for s-p53-antibody in the diagnosis of hepatocellular carcinoma. The point estimates of sensitivity/specificity from each study ar [file pone.0099255.s001.zip › supplementary materials--Dec/Table S1. .doc]

Table S1. Main characteristics of the 100 eligible studies.

a.

| First Author | Country | Ref. standard | Assay method^﹡^ | Cut-off | TP^﹡^ | FP^﹡^ | FN^﹡^ | TN^﹡^ |
| --- | --- | --- | --- | --- | --- | --- | --- | --- |
| Caron de Fromentel,C. | France | histology | 0 | unknown | 14 | 1 | 105 | 87 |
| Volkmann,M. | Germany | histology | 0 | unknown | 20 | 0 | 60 | 67 |
| Angelopoulou,K. | Canada | unknown | 0 | unknown | 61 | 1 | 1301 | 149 |
| Labrecque,S. | Canada | histology | 0 | unknown | 9 | 0 | 166 | 22 |
| Lahdeaho M.L. | Finland | unknown | 1 | unknown | 22 | 3 | 40 | 59 |
| Green J.A. | UK | unknown | 1 | unknown | 45 | 5 | 131 | 107 |
| Mudenda B. | UK | clinical diagnosis | 1 | unknown | 48 | 1 | 134 | 75 |
| Marxsen J. | Japan | histology | 1 | Formula 1^*^ | 5 | 2 | 73 | 80 |
| Lubin, R. | France | unknown | 1 | unknown | 112 | 1 | 1105 | 207 |
| Wild C. P. | France | histology | 1 | Formula 2^﹡^ | 16 | 0 | 170 | 50 |
| Green J. A. | UK | unknown | 1 | 5μg/ml | 11 | 1 | 27 | 72 |
| Laurent-Puig P. | UK | histology | 2 | unknown | 8 | 1 | 21 | 32 |
| Laurent-Puig P. | France | histology | 1 | unknown | 8 | 0 | 21 | 33 |
| Ryder,S.D. | Germany | unknown | 1 | unknown | 14 | 0 | 24 | 11 |
| Coomber D. | Sydney | unknown | 1 | unknown | 10 | 4 | 32 | 58 |
| Shibata Y. | Japan | unknown | 0 | Formula 3^﹡^ | 32 | 0 | 15 | 10 |
| Maass J.D. | Germany | unknown | 1 | Formula 4^﹡^ | 23 | 0 | 94 | 40 |
| Raedle J. | Germany | histology | 1 | unknown | 6 | 5 | 27 | 47 |
| Myrna R | Spain | histology | 0 | unknown | 27 | 0 | 143 | 50 |
| Angelopoulou K. | Canada | unknown | 0 | unknown | 53 | 2 | 176 | 228 |
| Hammel P. | France | histology | 1 | unknown | 14 | 0 | 40 | 24 |
| Friedrich, R.E. | Germany | unknown | 1 | unknown | 8 | 0 | 22 | 9 |
| Kaur J. | India | histology | 0 | unknown | 7 | 3 | 23 | 32 |
| Maass，J.D. | Germany | histology | 1 | Formula 5^﹡^ | 18 | 0 | 64 | 31 |
| Wollenberg, B. | Germany | histology | 1 | unknown | 37 | 27 | 60 | 113 |
| Wurl P. | Germany | unknown | 1 | unknown | 15 | 0 | 59 | 20 |
| Lai C.L. | Japan | unknown | 0 | unknown | 10 | 0 | 115 | 10 |
| Edis,C. | Innbrack | histology | 1 | unknown | 5 | 15 | 34 | 56 |
| Hallak R. | Germany | unknown | 1 | Formula 6^﹡^ | 42 | 3 | 193 | 191 |
| Ralhan,R. | India | unknown | 1 | unknown | 24 | 19 | 46 | 94 |
| Henlen M. | America | histology | 0 | unknown | 15 | 1 | 54 | 18 |
| Parashar K. | India | unknown | 1 | unknown | 6 | 0 | 14 | 20 |
| Shiota G | Japan | unknown | 1 | unknown | 8 | 0 | 17 | 10 |
| LANG C. | Germany | unknown | 1 | unknown | 13 | 1 | 169 | 15 |
| Sanchez-carbayo M. | Spanish | unknown | 1 | Index>1.1 | 14 | 0 | 45 | 15 |
| Schneider J. | Germany | bronchoscope | 1 | unknown | 22 | 21 | 94 | 158 |
| Schneider J. | Germany | histology | 1 | unknown | 19 | 9 | 59 | 64 |
| Saffory R. | France | histology | 1 | index>1.1 | 9 | 2 | 121 | 74 |
| Mayerhofer K. | Austria | unknown | 1 | Formula 7^﹡^ | 12 | 3 | 21 | 31 |
| Lenner P. | Sweden | unknown | 1 | unknown | 15 | 3 | 150 | 327 |
| Maehara Y | Japan | histology | 1 | index>1.1 | 23 | 0 | 97 | 15 |
| Wu C-W | USA | histology | 0 | unknown | 61 | 0 | 440 | 46 |
| Mayerhofer K | Austrialia | histology | 1 | unknown | 4 | 0 | 37 | 17 |
| Wunderlich H. | Germany | unknown | 1 | unknown | 4 | 0 | 28 | 10 |
| Rohayem J. | Germany | histology | 1 | unknown | 13 | 0 | 87 | 300 |
| Shimada H. | Japan | unknown | 1 | unknown | 84 | 2 | 150 | 206 |
| Mack U. | Germany | unknown | 1 | unknown | 17 | 1 | 117 | 45 |
| Feelay-Reynier I. | France | unknown | 1 | unknown | 2 | 1 | 19 | 19 |
| Metcalfe S. | Germany | histology | 1 | unknown | 21 | 1 | 92 | 116 |
| Shimada H. | Japan | histology | 1 | Formula 8^﹡^ | 14 | 0 | 21 | 69 |
| Hagiwara N. | Japan | histology | 1 | unknown | 13 | 0 | 33 | 13 |
| Ralhan R. | India | histology | 1 | unknown | 36 | 4 | 24 | 46 |
| Sakai H. | Japan | unknown | 1 | 1u/ml | 83 | 10 | 252 | 98 |
| Jassem E. | Poland | histology | 1 | Formula 8^﹡^ | 26 | 1 | 70 | 40 |
| Cioffi M. | Italy | unknown | 1 | unknown | 35 | 0 | 74 | 130 |
| Charuruks,N. | Thailand | AFP>400IU/L | 1 | unknown | 26 | 0 | 115 | 30 |
| Broll R. | Germany | histology | 1 | unknown | 20 | 0 | 110 | 44 |
| Tang R.P. | China Taiwan | histology | 1 | >10u/μl | 130 | 2 | 868 | 209 |
| Jezersek,B. | Slovenia | unknown | 1 | unknown | 67 | 4 | 41 | 16 |
| Numa F. | Japan | unknown | 1 | unknown | 8 | 0 | 22 | 9 |
| Kozlowski M. | Poland | unknown | 1 | Index>=1.1 | 20 | 0 | 55 | 10 |
| Chow,V. | China HK | histology | 1 | Formula 5^﹡^ | 23 | 1 | 52 | 27 |
| Wurl P | Germany | histology | 1 | unknown | 6 | 0 | 44 | 15 |
| Moch C. | France | histology | 1 | unknown | 3 | 2 | 102 | 128 |
| Hofele, C. | Germany | unknown | 1 | unknown | 19 | 0 | 83 | 80 |
| Estrid V.S. | Denmark | unknown | 1 | 10U/ml | 24 | 1 | 203 | 85 |
| Shimada H. | Japan | histology | 1 | 1.3U/ml | 28 | 7 | 77 | 146 |
| Ohshio，G. | Japan | histology | 1 | Formula 1^﹡^ | 19 | 1 | 63 | 20 |
| Shimada H. | Japan | unknown | 1 | 1.3U/ml | 221 | 23 | 864 | 371 |
| Neri M. | Italy | histology | 1 | unknown | 8 | 4 | 40 | 132 |
| Sangrajraing S. | Thailand | histology | 1 | unknown | 25 | 5 | 108 | 195 |
| Fonseca, R.F. | Brazil | unknown | 0 | unknown | 5 | 0 | 19 | 15 |
| Lee Y.F. | China | unknown | 1 | Formula 10^*^ | 17 | 0 | 260 | 79 |
| Lechpammer M. | America | histology | 1 | unknown | 40 | 0 | 180 | 42 |
| Wang M.H. | China | histology | 1 | Formula 8^﹡^ | 18 | 0 | 20 | 20 |
| Shaarawy M. | Egypt | histology | 1 | 100U/l | 48 | 0 | 24 | 20 |
| Megliorino R. | China | histology | 1 | Formula 10^*^ | 63 | 2 | 483 | 80 |
| Shi G.L. | China | histology | 1 | unknown | 61 | 0 | 109 | 98 |
| Himoto,T. | Japan | histology | 1 | Formula 10^*^ | 3 | 0 | 83 | 50 |
| Balogh G.A. | Multi center | histology | 1 | unknown | 9 | 0 | 46 | 8 |
| Hiroyuki K. | Japan | histology | 1 | 1.3 U/ml | 18 | 0 | 39 | 17 |
| Looi K. | China | unknown | 1 | Formula 10^*^ | 39 | 1 | 440 | 81 |
| Muller M. | Germany | histology | 0 | unknown | 138 | 0 | 453 | 436 |
| Sainger R.N. | India | histology | 1 | 0.85 U/ml | 17 | 13 | 58 | 102 |
| Akere A. | Nigeria | histology | 1 | unknown | 5 | 4 | 36 | 41 |
| Zhang,J.Y. | China | histology | 1 | Formula 10^*^ | 20 | 5 | 122 | 137 |
| Yashizawa S | Japan | histology | 1 | 5.85 U/100 μg | 43 | 44 | 39 | 305 |
| Al Kassam,D. | Spanish | histology | 2 | unknown | 13 | 0 | 26 | 10 |
| Lin-Lin Qiu Msc | China | histology | 1 | unknown | 16 | 0 | 45 | 30 |
| Mattionti M. | Italy | histology | 1 | unknown | 17 | 0 | 84 | 64 |
| C.J. Chapman | England | histology | 1 | Formula 10^*^ | 12 | 1 | 92 | 49 |
| Atta M.M. | Egypt | histology | 1 | Formula 10^*^ | 28 | 18 | 13 | 37 |
| Suppiah A. | England | histology | 1 | unknown | 20 | 0 | 72 | 28 |
| Cai H.Y. | China | histology | 1 | unknown | 18 | 0 | 28 | 30 |
| Attallah A.M. | Egypt | histology | 1 | Formula 10^*^ | 24 | 0 | 21 | 30 |
| Gadelhak N.A. | Egypt | unknown | 1 | 0.418 ng/ml | 38 | 3 | 29 | 34 |
| Wu M. | China | unknown | 1 | unknown | 83 | 9 | 486 | 870 |
| Anderson K.S. | America | unknown | 1 | 13.1×10^6^ | 28 | 13 | 62 | 137 |
| Kulic A. | Croatia | unknown | 1 | 15 U/ml | 21 | 1 | 40 | 19 |
| Park Y. | Korea | unknown | 1 | 2.7 U/ml | 28 | 1 | 54 | 23 |

Note: ELISA**^﹡^**=Enzyme-linked immunosorbent assay; TP**^﹡^**=true positives, FP**^﹡^**=false positives, FN**^﹡^**=false negatives, TN**^﹡^**=true negatives; Abs**^﹡^**= Antibody.

Assay method^﹡^：ELISA or ELISA+western=1; other=0; unknown=2.

Formula 1^﹡^: normal+2SD

Formula 2^﹡^: P53 control protein ratio >1.3

Formula 3^﹡^: density>1030

Formula 4^﹡^: optical density>0.11

Formula 5^﹡^: manufacturer’s formula

Formula 6^﹡^: the differences in the absorption values between the positive walls and controls was greater than 0.5

Formula 7^﹡^: differences higher than 0.15 were scored positive

Formula 8^﹡^: index≥1.1,Absorbtion>=1.6

Formula 9^*^ : ≥2.3-times of control

Formula 10^*^ : mean+3SD

b

| Author | Consecutive/random | Histology  Well/Moderate/poorly/other | Sample collection time^*^ | Stage I(%) | QUADAS |
| --- | --- | --- | --- | --- | --- |
| Caron de Fromentel,C. | unknown | unknown | 2 | unknown | 8 |
| Volkmann,M. | unknown | unknown | 2 | unknown | 7 |
| Angelopoulou,K. | unknown | unknown | 2 | unknown | 7 |
| Labrecque,S. | unknown | unknown | 2 | unknown | 7 |
| Lahdeaho M.L. | unknown | unknown | 2 | unknown | 6 |
| Green J.A. | Consecutive | unknown | 2 | unknown | 5 |
| Mudenda B. | unknown | unknown | 2 | unknown | 8 |
| Marxsen J. | unknown | unknown | 2 | 5/82 6.10% | 8 |
| Lubin, R. | unknown | unknown | 0 | unknown | 7 |
| Wild C. P. | Consecutive | unknown | 0 | unknown | 10 |
| Green J. A. | unknown | unknown | 2 | unknown | 6 |
| Laurent-Puig,P. | unknown | unknown | 2 | unknown | 7 |
| Laurent-Puig,P. | unknown | unknown | 0 | unknown | 9 |
| Ryder,S.D. | unknown | unknown | 2 | unknown | 7 |
| Coomber D. | unknown | unknown | 2 | unknown | 6 |
| Shibata Y. | unknown | unknown | 2 | unknown | 6 |
| Maass，J.D. | unknown | unknown | 2 | unknown | 5 |
| Raedle,J. | Consecutive | unknown | 2 | unknown | 9 |
| Myrna R | unknown | unknown | 2 | unknown | 7 |
| Angelopoulou K. | unknown | 12/128/11 | 2 | 23/229 10.04% | 6 |
| Hammel, P. | unknown | unknown | 0 | 10/54 18.52% | 9 |
| Friedrich, R.E. | unknown | unknown | 2 | 1/30 3.33% | 6 |
| Kaur, J. | unknown | 14/11/5 | 2 | unknown | 9 |
| Maass，J.D. | unknown | unknown | 0 | unknown | 8 |
| Wollenberg, B. | unknown | 14/35/24 | 2 | 16/82 19.51% | 9 |
| WURL P. | unknown | 3/16/55 | 0 | 11/74 14.86% | 9 |
| Lai C.L. | Consecutive | unknown | 2 | 11/125 9.00% | 6 |
| Edis,C. | unknown | unknown | 2 | unknown | 8 |
| Hallak R. | unknown | health | 2 | unknown | 6 |
| Ralhan,R. | unknown | unknown | 2 | unknown | 8 |
| Henlen M. | unknown | unknown | 2 | unknown | 8 |
| Parashar K. | unknown | unknown | 2 | unknown | 6 |
| Shiota G | unknown | 10/7/2 | 0 | 5/25 20.00% | 9 |
| LANG C. | unknown | unknown | 2 | unknown | 7 |
| Sanchez-carbayo M. | unknown | 21/15/23 | 2 | unknown | 6 |
| Schneider J. | unknown | unknown | 2 | unknown | 9 |
| Schneider J. | unknown | unknown | 2 | unknown | 9 |
| Saffory R. | Consecutive | unknown | 0 | 6/130 4.61% | 11 |
| Mayerhofer K. | unknown | 7/4/4/4/5 | 2 | 8/33 24.24% | 9 |
| Lenner P. | Consecutive | unknown | 0 | 69/165 41.82% | 6 |
| Maehara Y | unknown | 63/57 | 0 | unknown | 9 |
| Wu C-W | unknown | unknown | 2 | unknown | 7 |
| Mayerhofer K | unknown | 17/24 | 0 | 19/41 46.34% | 9 |
| Wunderlich H. | unknown | unknown | 2 | unknown | 6 |
| Rohayem J. | unknown | unknown | 2 | unknown | 7 |
| Shimada H. | unknown | unknown | 2 | unknown | 5 |
| Mack U. | unknown | unknown | 2 | 11/134 8.21% | 6 |
| Feelay-Reynier I. | unknown | unknown | 2 | unknown | 7 |
| Metcalfe S. | unknown | unknown | 0 | 23/113 20.35% | 10 |
| Shimada H. | Consecutive | 7/16/10/0 | 0 | unknown | 10 |
| Hagiwara N. | unknown | unknown | 0 | 6/46 13.0% | 7 |
| Ralhan R. | unknown | 26/20/12/0 | 0 | 6/60 10.0% | 9 |
| Sakai H. | unknown | unknown | 2 | unknown | 6 |
| Jassem E. | Consecutive | unknown | 2 | unknown | 8 |
| Cioffi M. | unknown | unknown | 2 | 13/109 11.93% | 5 |
| Charuruks,N. | unknown | unknown | 2 | 4/141 2.83% | 6 |
| Broll R. | Consective | 7/95/28/0 | 1 | 41/130 31.54% | 9 |
| Tang R.P. | unknown | 195/725/78 | 2 | unknown | 9 |
| Jezersek,B. | unknown | unknown | 1 | unknown | 7 |
| Numa F. | unknown | unknown | 2 | unknown | 8 |
| Kozlowski M. | Consecutive | 14/27/34 | 1 | 4/75 5.3% | 7 |
| Chow,V. | unknown | unknown | 2 | 19/75 25.3% | 9 |
| Peter Wurl | unknown | unknown | 0 | 10/50 20% | 9 |
| Moch C. | unknown | unknown | 2 | unknown | 8 |
| Hofele, C. | unknown | unknown | 2 | unknown | 7 |
| Estrid V.S. | unknown | 13/110/70 | 2 | 50/227 22.03% | 7 |
| Shimada H. | Consecutive | unknown | 2 | 50/105 47.6% | 8 |
| Ohshio，G. | unknown | unknown | 2 | 5/82 6.10% | 9 |
| Shimada H. | unknown | unknown | 2 | unknown | 6 |
| Neri M. | unknown | unknown | 2 | 2/48 4.20% | 8 |
| Sangrajraing S. | unknown | unknown | 2 | 2/133 1.50% | 7 |
| Fonseca, R.F. | unknown | unknown | 2 | unknown | 8 |
| Lee Y.F. | Consecutive | unknown | 2 | 7/277 2.53% | 5 |
| Lechpammer M. | unknown | 100% adenocarcinoma | 0 | 28/220 12.70% | 9 |
| Wang M.H. | unknown | 12/12/14/0 | 0 | 10/38 26.30% | 9 |
| Shaarawy M. | unknown | unknown | 2 | unknown | 8 |
| Megliorino R. | unknown | unknown | 0 | unknown | 8 |
| Shi G.L. | unknown | unknown | 1 | unknown | 10 |
| Himoto,T. | random | unknown | 2 | unknown | 9 |
| Balogh G.A. | unknown | unknown | 2 | 17/55 30.91% | 8 |
| Hiroyuki K. | unknown | 13/19/17/8 | 2 | 13/57 22.80% | 8 |
| Looi K. | unknown | unknown | 1 | unknown | 7 |
| Muller M. | Consecutive | unknown | 2 | unknown | 7 |
| Sainger R.N. | unknown | 21/54 | 2 | 5/75 6.67% | 9 |
| Akere A. | Consecutive | unknown | 2 | unknown | 8 |
| Zhang,J.Y. | unknown | unknown | 1 | 51/142 35.92% | 9 |
| Yashizawa S | unknown | unknown | 2 | unknown | 9 |
| Al Kassam,D. | unknown | unknown | 2 | 2/39 5.13% | 7 |
| Lin-Lin Qiu Msc | unknown | unknown | 2 | 5/61 8.12% | 8 |
| Mattionti M. | unknown | unknown | 2 | 20/111 18.02% | 9 |
| C.J. Chapman | unknown | unknown | 2 | 5/104 4.80% | 8 |
| Atta M.M. | unknown | unknown | 2 | 13/41 31.70% | 10 |
| Suppiah A. | unknown | unknown | 1 | 5/92 5.43% | 10 |
| Cai H.Y. | Consecutive | 15/17/14/0 | 0 | 10/46 21.70% | 8 |
| Attallah A.M. | unknown | unknown | 2 | unknown | 8 |
| Gadelhak N.A. | Random | unknown | 2 | unknown | 8 |
| Wu M. | Random | unknown | 2 | unknown | 6 |
| Anderson K.S. | unknown | unknown | 2 | unknown | 8 |
| Kulic A. | unknown | unknown | 2 | unknown | 6 |
| Park Y. | unknown | unknown | 2 | unknown | 7 |

Note: Sample collection time*: before diagnosis=1; before treament=0; unknown=2
