# Supplementary material for: Assessment of the Potential Diagnostic Value of Serum p53 Antibody for Cancer: A Meta-Analysis
Source: PLoS One. 2014 Jun 9;9(6):e99255. doi: 10.1371/journal.pone.0099255 (PMC4049633; doi:10.1371/journal.pone.0099255)
Supplement: File S1 — Supporting figures, tables and references. Figure S1 Methodological quality graph: review authors' judgments about each methodological quality item presented as percentages across all included studies. (PDF). Figure S2 Forest plot of sensitivity and specificity of 5 individual studies for s-p53-antibody in the diagnosis of bladder cancer. The point estimates of sensitivity/specificity from each study are shown as solid circles. Error bars are 95% confidence intervals. Figure S3 Forest plot of sensitivity and specificity of 12 individual studies for s-p53-antibody in the diagnosis of breast cancer. The point estimates of sensitivity/specificity from each study are shown as solid circles. Error bars are 95% confidence intervals. Figure S4 Forest plot of sensitivity and specificity of 16 individual studies for s-p53-antibody in the diagnosis of colorectal cancer. The point estimates of sensitivity/specificity from each study are shown as solid circles. Error bars are 95% confidence intervals. Figure S5 Forest plot of sensitivity and specificity of 15 individual studies for s-p53-antibody in the diagnosis of EC. The point estimates of sensitivity/specificity from each study are shown as solid circles. Error bars are 95% confidence intervals. Figure S6 Forest plot of sensitivity and specificity of 6 individual studies for s-p53-antibody in the diagnosis of gastric cancer. The point estimates of sensitivity/specificity from each study are shown as solid circles. Error bars are 95% confidence intervals. Figure S7 Forest plot of sensitivity and specificity of 7 individual studies for s-p53-antibody in the diagnosis of head and neck cancer. The point estimates of sensitivity/specificity from each study are shown as solid circles. Error bars are 95% confidence intervals. Figure S8 Forest plot of sensitivity and specificity of 17 individual studies for s-p53-antibody in the diagnosis of hepatocellular carcinoma. The point estimates of sensitivity/specificity from each study ar [file pone.0099255.s001.zip › supplementary materials--Dec/Table S2.doc]

## Additional information

## Table S2 Search strategy in PubMed

| Search | Query | Items found |
| --- | --- | --- |
| #1 | Neoplasms [Mesh] | 2379279 |
| #2 | Tumor OR tumour OR neoplasm OR neoplasms OR cancer | 2955877 |
|  | OR melanoma OR carcinoma [all fields] |  |
| #3 | #1 or #2 | 2955877 |
| #4 | P53 or TP53 [all fields] | 64412 |
| #5 | Antibody or autoantibody [all fields] | 1084340 |
| #6 | #4 and #5 | 6339 |
| #7 | Blood OR serum OR serological OR seropositive OR | 3882424 |
|  | seropositivity OR serum antibody OR sera OR plasma[all fields] |  |
| #8 | #3 and #6 and #7 limits: humans | 1090 |

Note: Database: PubMed. Search time limits : May 31st, 2012
